# Humoral immune response to tumor-associated antigen Ubiquilin 1 (UBQLN1) and its tumor-promoting potential in lung cancer

**DOI:** 10.1186/s12885-024-12019-w

**Published:** 2024-03-02

**Authors:** Yulin Wang, Songyun Ouyang, Man Liu, Qiufang Si, Xue Zhang, Xiuzhi Zhang, Jiaqi Li, Peng Wang, Hua Ye, Jianxiang Shi, Chunhua Song, Kaijuan Wang, Liping Dai

**Affiliations:** 1 Henan Institute of Medical and Pharmaceutical Sciences & Henan Key Medical Laboratory of Tumor Molecular Biomarkers, Zhengzhou University, Zhengzhou, Henan, 450052 China; 2 Henan Key Laboratory of Tumor Epidemiology, Zhengzhou University, Zhengzhou, Henan, 450052 China; 3 Department of Respiratory and Sleep Medicine, the First Affiliated Hospital of Zhengzhou University, Zhengzhou, Henan, 450052 China; 4 Laboratory of Molecular Biology, Henan Luoyang Orthopedic Hospital (Henan Provincial Orthopedic Hospital), Zhengzhou, China

**Keywords:** Pulmonary nodules, Autoantibody, UBQLN1, Diagnostic model, Lung cancer

## Abstract

**Background:**

This study aims to investigate the expression of UBQLN1 in lung cancer (LC) tissue and the diagnostic capability of autoantibody to UBQLN1 (anti-UBQLN1) in the detection of LC and the discrimination of pulmonary nodules (PNs).

**Methods:**

Sera from 798 participants were used to discover and validate the level of autoantibodies via HuProt microarray and Enzyme-linked immunosorbent assay (ELISA). Logistic regression analysis was applied to establish model. Receiver operating characteristic curve (ROC) analysis was performed to evaluate the diagnostic potential. Immunohistochemistry was performed to detect UBQLN1 expression in 88 LC tissues and 88 para-tumor tissues. qRT-PCR and western blotting were performed to detect the expression of UBQLN1 at the mRNA and protein levels, respectively. Trans-well assay and cell counting kit-8 (CCK-8) was used to investigate the function of UBQLN1.

**Results:**

Anti-UBQLN1 was identified with the highest fold change by protein microarray. The level of anti-UBQLN1 in LC patients was obviously higher than that in NC or patients with benign lung disease of validation cohort 1 (*P*<0.05). The area under the curve (AUC) of anti-UBQLN1 was 0.610 (95%CI: 0.508-0.713) while reached at 0.822 (95%CI: 0.784-0.897) when combining anti-UBQLN1 with CEA, CYFRA21-1, CA125 and three CT indicators (vascular notch sign, lobulation sign and mediastinal lymph node enlargement) in the discrimination of PNs. UBQLN1 protein was overexpressed in lung adenocarcinoma (LUAD) tissues compared to para-tumor tissues. UBQLN1 knockdown remarkably inhibited the migration, invasion and proliferation of LUAD cell lines.

**Conclusions:**

Anti-UBQLN1 might be a potential biomarker for the diagnosis of LC and the discrimination of PNs.

**Supplementary Information:**

The online version contains supplementary material available at 10.1186/s12885-024-12019-w.

## Background

Lung cancer (LC) is one of the most common malignant tumors and the leading cause of cancer-related death worldwide [1]. It is estimated that approximately 2.2 million new LC cases and 1.8 million deaths occur in 2020, which represents 11.4% of total new cancer cases and 18.0% of total new deaths, respectively [1]. The 5-year survival rate for metastatic LC is 6% while it could be up to 57% for localized cases [2]. Numerous evidences showed that low-dose computed tomography (LDCT), as a powerful means, can be used for screening high-risk populations at an early stage and thus improve the survival rate of LC patients [1, 3]. Due to its high false positive ratio, it is of necessity to combine this approach with other diagnostic methods to achieve higher diagnostic capacity.

In the 1960s, Robet W. Baldwin found that several physiological processes such as specific point mutations, misfolding, overexpression, aberrant glycosylation, truncation or aberrant degradation could lead to the abnormal expression of some proteins, these proteins were known as tumor associated antigens (TAAs) [4]. TAAs can elicit immune responses and stimulate autoantibodies against TAAs (TAAbs) before or during tumor formation [5]. Over the past few decades, TAAbs have been considered as a group of potential biomarkers because of its stability and ease of detection [5, 6].

To date, numerous technologies such as serological analysis of recombination cDNA expression libraries (SEREX), serological proteome analysis (SERPA) and protein microarray were utilized to discover novel TAAbs. Our previous studies have discovered several novel TAAbs of LC based on different screening approaches and further proved that the combination of TAAbs and other traditional biomarkers can dramatically improve the diagnostic accuracy of LC [7–11].

The family of UBQLN1 has been reported to be involved in regulating the process of endoplasmic reticulum-associated protein degradation (ERAD) and the occurrence of some neurological disorders [12]. UBQLN1 as an essential factor is related to several biological processes such as ERAD [13], epithelial to mesenchymal transition (EMT) [14] and neurodegeneration [15]. Besides, Chen and his colleagues have proved that antibodies against UBQLN1 (anti-UBQLN1) can be used for screening lung adenocarcinoma (ADC) patients from healthy individuals [16]. However, whether anti-UBQLN1 can discriminate malignant pulmonary nodules (MPN) from benign pulmonary nodules (BPN) patients is unclear.

In the present study, we use two independent cohorts to validate the diagnostic capability of anti-UBQLN1 in the detection of LC patients and discrimination of pulmonary nodules. Furthermore, we also investigated the expression of UBQLN1 in LC and paracancerous tissues by tissue chip and explored the role of UBQLN1 in promoting the development of LC in vitro.

## Materials and methods

### Study population and serum samples collection

A total of 798 serum samples were collected from the First Affiliated Hospital of Zhengzhou University (Zhengzhou, Henan). 212 LC patients, 144 BLD patients and 210 PN patients were recruited from November 2016 to November 2019 at the time of initial diagnosis without any treatment. 212 NC were recruited from medical examination population from May 2019 to June 2019. Five milliliter peripheral blood sample was drawn and separated by centrifugation at 3000 rpm for 5 min and then stored at -80 ℃ for further experiments.

This study was conducted in accordance with the Declaration of Helsinki. The study protocol was permitted by the Ethics Committee at Zhengzhou University and all the participants have signed informed consent. The concentrations of CEA, CYFRA211 and CA125 in serum were detected by electro-chemiluminescence immunoassay (Roche, USA). 12 CT characteristics (number, diameter, edge, spiculation, vascular notch sign, lobulation, spines, pleural indentation, mediastinal lymph node enlargement, emphysema and calcification) were judged by two radiologists.

### Huprot protein microarray assay

HuProt^TM^ v3.1 protein microarray was purchased from BCBIO technology (Guangzhou, China). Protein microarray was applied to screen the candidate autoantibody for the diagnosis of LC. Initially, the microarray need to be removed from the -80℃ refrigerator and then blocked by 3% BSA at room temperature for 1 h before incubation. Subsequently, the microarray was incubated with serum sample (dilution: 1:200) as primary antibody at 4℃ overnight. After washing with PBST, the microarray was incubated with 1:1000 dilution of secondary antibody at room temperature for 1h in the dark. After washing with PBST and ddH_2_O, the microarray was dried and then scanned with LuxScan 10K-A (CapitalBio).

The medians of foreground (F532 Median) and background (B532 Median) intensity of each protein at 532 nm were observed by scanning instrument. The ratio of F532 Median to B532 Median was F/B defined as SNR for the normalization of microarray. The normalization among microarrays was conducted by z-score. The positive ratio of anti-UBQLN1 in LC or NC refers to the ratio of the number whose SNR are higher than 6.238 (cutoff) to the total in LC or NC group. The analysis method for screening candidate protein was as follows: The TAAb with maximum fold change (FC: the ratio of median of LC to NC) was consider as the candidate.

### Enzyme-Linked Immunosorbent Assay (ELISA)

The level of anti-UBQLN1 was detected by ELISA, followed by our previous research [8, 10, 17]. Commercial purified recombinant UBQLN1 protein was purchased from CUSABIO technology (Wuhan, Hubei). Initially, UBQLN1 protein with a concentration of 0.125 μg/ml was coated to 96-well plates (50μg/well) at 4℃ and then blocked in 2% bovine serum albumin (100 μg/well, BSA) (Solaibio, Beijing) at 4℃ overnight. Subsequently, the plates washed by phosphate-buffered saline (PBS) containing 0.05 % Tween 20 (PBST) were incubated with a 1:100 dilution of sera as primary antibody and a 1:10000 dilution of horseradish peroxidase (HRP) labeled mouse anti-human secondary antibodies (Santa Cruz, USA) as secondary antibody at 37℃. TMB (Solaibio, Beijing) and 10% H_2_SO_4_ were used as the detection reagent and stop solution, respectively. The optical density (OD) value was read at 405 nm by enzyme micro-plate reader. All LC, NC, BLD, MPN and BPN sera samples were randomly dispensed on the plates. Blank control was set on each plate for ensuring the stability of assay.

### Western Blotting (WB)

Western blotting was used to confirm the expression of anti-UBQLN1 in serum samples. The detailed information was provided as follows: (a) Purified UBQLN1 protein was loaded onto 10% polyacrylamide gelelectrophoresis (SDS-PAGE) (Leagene, Beijing) and transferred onto polyvinylidene fluoride (PVDF) membranes (Millipore, USA). (b) After blocking with 5% nonfat milk (Solaibio, Beijing) in TBST for 2h, membranes were incubated with 1:200 dilution of representative serum samples and 1:5000 dilution of HRP labeled mouse anti-human secondary antibodies (Santa Cruz, USA). (c) Blotting results were detected by electrochemiluminescence (ECL) chemiluminescence kit (Thermo, USA).

In the present study, western blotting was also performed to detect protein from cell lines. The detailed information was provided as follows: (a) Total protein from different cell lines was extracted by RIPA buffer (Solaibio, Beijing) which is used to lyse cells and contains protease inhibitor. (b) Protein concentration was measured by Bicinchoninic acid (BCA) kit (Solaibio, Beijing). (c) Protein samples was loaded onto 10% polyacrylamide gelelectrophoresis (SDS-PAGE) (Leagene, Beijing) and transferred onto polyvinylidene fluoride (PVDF) membranes (Millipore, USA). (d) After blocking with 5% nonfat milk (Solaibio, Beijing) in TBST for 2h, membranes were incubated with 1:5000 dilution of primary antibodies (NBP1-56536, Novus, USA) and 1:5000 dilution of horseradish peroxidase (HRP) labeled goat anti-human secondary antibodies (ZENBIO, Chengdu). (e) The results were detected by electrochemiluminescence (ECL) chemiluminescence kit (Thermo, USA).

### Cell Culture

Beas-2b (B2B), H1299, PC-9 and H1975 cell lines were purchased from American Type Culture Collection (ATCC, USA). CALU-3 cell line was obtained from Jennio biological technology (Guangzhou, China). A549 and H358 cell lines were procured from the Cell Bank of the Chinese Academy of Sciences (Shanghai, China). Of the seven cell lines, five (PC-9, H1975, H1299, A549 and H358) were cultured in 1640 medium (BI, Israel) supplemented with 10% fetal bovine serum (FBS) while B2B and CALU-3 cells were maintained in DMEM medium (BI, Israel) supplemented with 10% FBS.

### RNA Extraction and Quantitative RT-PCR (qRT-PCR)

Total RNA from different cell lines was extracted using TRIZOL regent (Takara, Japan) and then was reverse-transcribed to cDNA using reverse transcription kit (RR047A)(Takara, Japan), followed by supplier’s protocol. qRT-PCR for UBQLN1 was carried out using TB Green® Premix Ex Taq™II (RR820) and (Takara, Japan) and an ABI Q3 system (Applied Biosystems, CA) according to manufacturer’s instructions. GAPDH was served as a stable endogenous control for normalization. GAPDH and UBQLN1 primers were from SYBR Green qRT-PCR primer set (Sangon, Shanghai). The primer sequences were provided as follows:

- GAPDH-F: CAGGAGGCATTGCTGATGAT
- GAPDH-R: GAAGGCTGGGGCTCATTT
- UBQLN1-F: GCCAATCCACAAATGCAGCAGTTG
- UBQLN1-R: TCGGTCCTGGTTCCTCATCATCTC

### Cell Transfection

Three UBQLN1 siRNA (si-U1, si-U2, si-U3) and negative controls were purchased from RiboBio technology (RiboBio, China). Cells (2.5×10^5^ /well) were seeded into six-well plates (Corning, USA) and cultured. In the following day, siRNA and transfection reagent (Lipofectamine® 3000 Reagent, Invitrogen, USA) were mixed and then added to the plates.

### Cell migration and invasion assay in vitro

Transwell assay was used to check cell migration and invasion. Two cell lines (H358 and CALU-3) expressing UBQLN1 siRNA (si-U1, si-U3) and negative controls were seeded into 96-well plates (2-3×10^3^cell/well) and supplemented with corresponding transfection reagents after 6-8h. Six replicates in each group were used to ensure the stability of experiment.

### Cell proliferation assay in vitro 

Cell Counting Kit-8 (CCK-8) assay was performed to evaluate cell proliferation by CCK-8 kit (Meilunbio, Dalian). Two cell lines expressing UBQLN1 siRNA (si-U1, si-U3 and NC) (2-3×10^3^cell/well) were seeded into 96-well plates (Corning, USA) and supplemented with corresponding transfection reagents after 6-8h. Six replicated wells in each group were used to ensure the stability of experiment. CCK-8 buffer (CCK-8: DMED/1640=1:9, 100 μL/well) was added into wells with transfected cells cultured at 37℃ for 24, 48, 72 and 96 h. The OD value was measured at 450 nm.

### Immunohistochemistry (IHC)

The tissue microarray including 88 LC tissues and 88 para-cancerous tissues used for the analysis of UBQLN1 were purchased from Outdo Biotech (Shanghai, China). Anti-UBQLN1 antibody (NBP1-56536, Novus, USA) at 1:100 dilutions was used as primary antibody for IHC. The results of IHC were judged by two experienced pathologists. In brief, the staining intensity was graded as 0 (negative, -), 1 (weak, +), 2 (moderate, ++), and 3 (strong, +++), and the percentage of staining cells was scored as 0 (<5%), 1 (5–25%), 2 (26–50%), 3 (51–75%), and 4 (76–100%). The value that multiplying staining intensity and the percentage of staining cells was considered as total score. The total score > 6 and ≤6 was considered as high expression and low expression, respectively.

### Statistical analysis

All data were visualized by SPSS 25, GraphPad Prism 8.0 and Image Scope. The level of anti-UBQLN1 between two groups was analyzed by Manu-Whitney U Test. χ^2^ test and Fisher’s Exact Test were employed to compare the differences of 12 CT indicators between MPN and BPN patients and the expression of UBQLN1 protein among tissues from ADC patients with different clinical features. Logistic regression analysis was performed to establish model for the discrimination of PN patients. The receiver operating characteristic (ROC) curve analysis was applied to evaluate the diagnostic ability of anti-UBQLN1 and model in different group populations. The predictive value with the maximum Youden’s index (YI) was considered as the cutoff for the discrimination model.

## Results

### Study design

**Fig. 1:**
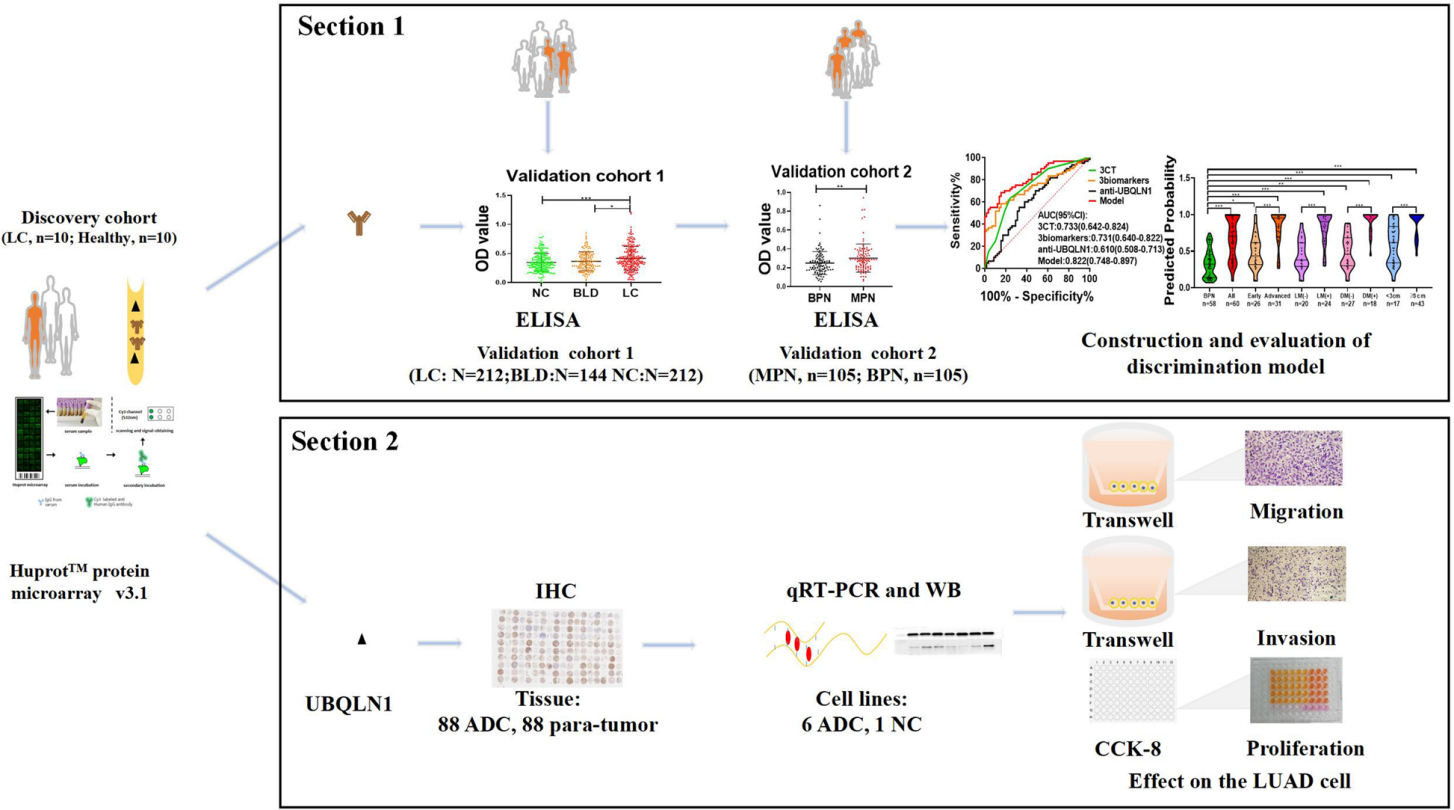
Schematic diagram of identification of UBQLN1 based on Huprot protein microarray. NC: normal control, BLD: benign lung disease, LC: lung cancer, MPN: malignant pulmonary nodule, BPN: benign pulmonary nodule, LUAD: lung adenocarcinoma. WB: western blotting, ELISA: Enzyme-linked immunosorbent assay, IHC: immunohistochemistry.

The design of current study was showed in Fig. 1. Firstly, sera from 10 LC patients and 10 healthy individuals were respectively used as a sera pool to screen candidate autoantibodies based on protein microarray and discover anti-UBQLN1 as the candidate. Later, two sections were carried out to determine the performance of anti-UBQLN1 in the diagnosis of LC and explore the function of UBQLN1 as a TAA.

Table 1 presented the characteristics of total participants. Three independent cohorts (discovery cohort, validation cohort 1 and validation cohort 2) were used in this study. In discovery cohort, 10 LC sera and 10 NC sera matched by age and gender were detected through protein microarray. Serum samples from 212 LC patients, 212 matched NC, 144 benign lung diseases (BLD) patients, as well as 105 malignant pulmonary nodule (MPN) and 105 benign pulmonary nodule (BPN) patients were involved in validation cohort 1 and validation cohort 2, respectively.

**Table 1:** Characteristic of participants in discovery cohort, validation cohort 1 and validation cohort 2

	Discovery cohort				Validation cohort 1				Validation cohort 2		
	LC	NC	*P*	LC		BLD	NC	*P*	MPN	BPN	*P*
N	10	10		212		144	212		105	105	
*Age(y)*			0.280					0.296			0.643
Mean±SD	63±12	57±10		59±12		60±10	56±12		55±9	57±9	
Range	43-82	39-70		26-85		29-85	28-89		26-72	31-81	
*Gender*			1.0					1.0			1.0
Male (%)	6(60.0)	6(60.0)		110(51.9)		103(71.5)	110(51.9)		62(59.0)	62(59.0)	
Female (%)	4(40.0)	4(40.0)		102(48.1)		41(28.5)	102(48.1)		43(41.0)	43(41.0)	
*Smoking*											0.796
Yes (%)				96(45.3)		78(54.2)			32(30.5)	30(28.6)	
No (%)				104(49.1)		66(45.8)			65(61.9)	66(62.8)	
Unknown (%)				12(5.6)		0(0.0)			8(7.6)	9(8.6)	
*Drinking*											0.560
Yes (%)				52(24.5)		36(25.0)			17(16.2)	20(19.0)	
No (%)				148(69.8)		108(75.0)			80(76.2)	76(72.4)	
Unknown (%)				12(5.7)		0(0.0)			8(7.6)	9(8.6)	
*Clinical stage*											
I (%)	3(30.0)			36(17.0)					30(28.6)		
II (%)	1(10.0)			15(7.1)					14(13.3)		
III (%)	3(30.0)			69(32.5)					15(14.3)		
IV (%)	3(30.0)			79(37.3)					34(32.4)		
Unknown (%)				13(6.1)					12(11.4)		
*Histologic type*											
*COPD*(%)						72(50.0)				2(2.0)	
*CB*(%)						72(50.0)				85(81.0)	
*NSCLC*											
ADC (%)	6(60.0)			71(33.5)					69(65.7)		
SCC (%)	4(40.0)			95(44.8)					14(13.3)		
LCLC (%)				2(0.9)					1(1.0)		
*SCLC*				34(16.0)					6(5.7)		
*Unknown (%)*				10(4.7)					15(14.3)	18(17.0)	
*Lymph node Metastasis*											
Yes (%)				131(61.7)					49(46.7)		
No (%)				68(32.2)					44(41.9)		
Unknown (%)				13(6.1)					12(11.4)		
*Distant metastasis*											
Yes (%)				80(37.7)					34(32.4)		
No (%)				119(56.2)					59(56.2)		
Unknown (%)				13(6.1)					12(11.4)		
*CEA*								0.001^*^			0.001^*^
Median±IQR				3.22±7.30			2.11±1.67		2.68±5.51	1.70±2.07	
0−5 ng/mL(%)				80(37.7)			187(88.2)		56(53.3)	62(59.0)	
>5 ng/mL (%)				42(19.8)			11(5.2)		23(21.9)	4(3.8)	
Unknown (%)				90(42.5)			14(6.6)		26(24.8)	39(37.2)	
*CA125*											0.016^*^
Median±IQR									17.40±49.19	11.31±19.07	
0-35 U/ml (%)									44(41.9)	52(49.5)	
>35 U/ml (%)									27(25.7)	11(10.5)	
Unknown (%)									34(32.4)	42(40.0)	
*CYFRA211*											0.02^*^
Median±IQR									2.24±4.39	1.85±0.93	
0-3.3 ng/mL (%)									46(43.8)	54(51.4)	
>3.3 ng/mL (%)									30(28.6)	7(6.7)	
Unknown (%)									29(27.6)	44(41.9)	

*NC* normal control, *BLD* benign lung disease, *LC* lung cancer, *MPN* malignant pulmonary nodule, *BPN* benign pulmonary nodule, *SD* standard deviation, *ADC* adenocarcinoma, *SCC* squamous cell carcinoma, *NSCLC* non-small cell lung cancer, *SCLC* small cell lung cancer, *COPD* chronic obstructive pulmonary disease, *CB* chronic bronchitis

Section 1 includes (a) sera from 212 LC patients, 144 BLD patients and 212 NC was used to test the level of anti-UBQLN1 in validation cohort 1; (b) 210 PN patients’ samples were utilized to detect the expression of anti-UBQLN1 in validation cohort 2; (c) 118 PN patients with the results of CEA, CYFRA211, CA125 and 12 CT indicators was applied to construct the discrimination model of PN patients and evaluate the diagnostic ability of this model in patients with several clinical characteristics.

In Section 2: (a) the expression of UBQLN1 protein was detected in 88 lung ADC tissues and 88 adjacent normal tissues. The detailed information from tissue samples was showed in Table S1; (b) seven cell lines were used to detect the expression of UBQLN1 at mRNA and protein level; (c) Two cell lines (CALU3 and H358) transfected with two types of siRNA were used to investigate the function of UBQLN1 in LC cell lines.

### Anti-UBQLN1 was identified as a candidate biomarker in lung cancer based on protein microarray

An increasing number of researches proved that Huprot microarray as a kind of powerful technique has been widely applied for the identification of biomarker and drug target [18–20]. Hence, we utilized it to screen the candidate biomarker and the corresponding antibody [19]. The anti-UBQLN1 with the highest FC of 6.93 and *P*<0.05, was screened via protein microarray and it showed a high sensitivity of 50% and specificity of 90%. Fig. S1 exhibited the specific SNR of UBQLN1 in 20 individuals. It can be seen from Fig. S1 that the SNR of UBQLN1 in lung cancer group is obviously higher than that in healthy individuals.

### The level of anti-UBQLN1 in LC patients was higher than that in NC and BLD patients

**Fig 2:**
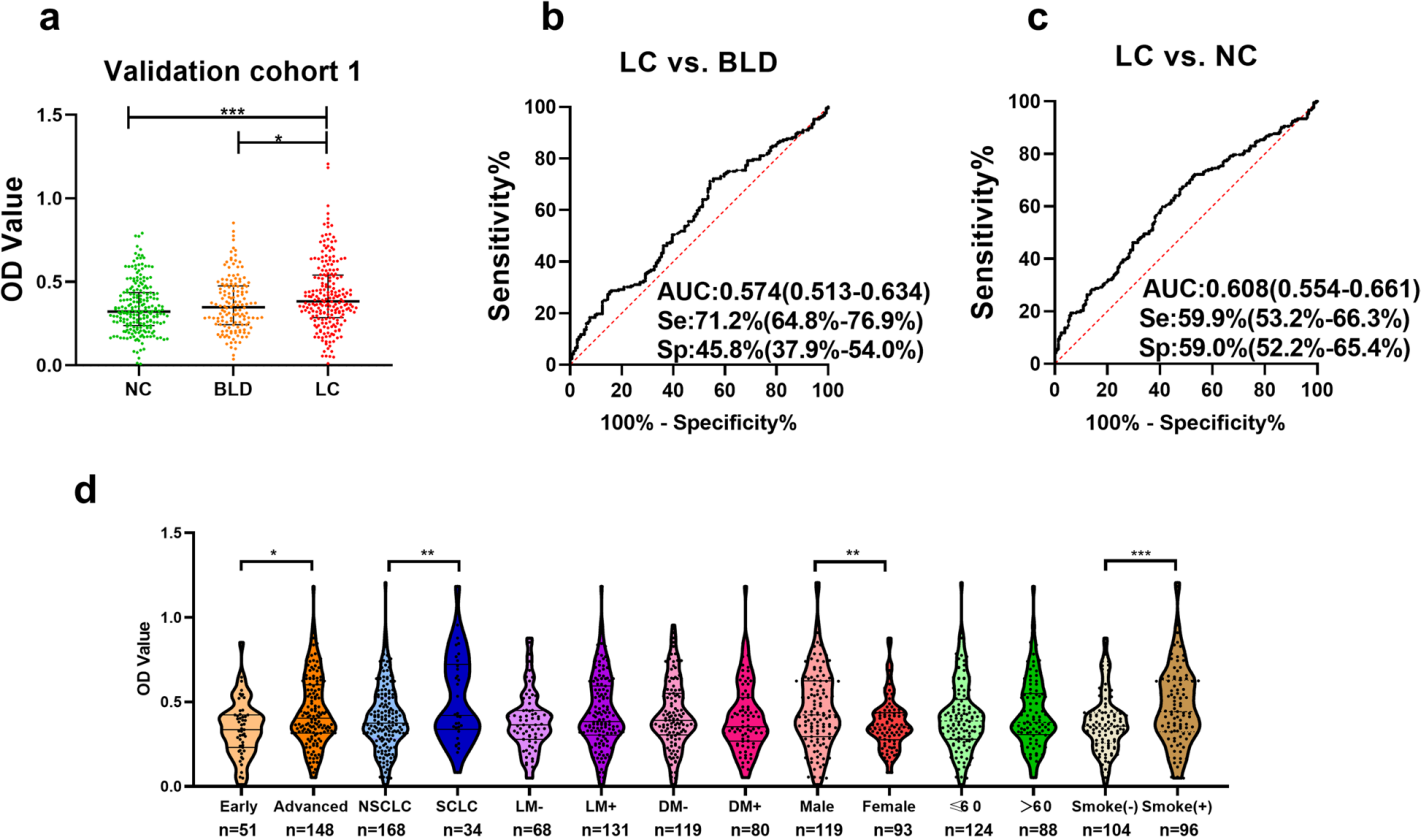
Diagnostic performance of anti-UBQLN1 in validation cohort 1 a. The scatter plot of OD value of anti-UBQLN1 in validation cohort 1. b-c. ROC of anti- UBQLN1 for the discrimination between LC and NC, LC and BLD. d. violin plot and scatter plot of OD value of anti-UBQLN1 in different subgroup of validation cohort 1. NC: normal control, BLD: benign lung disease, LC: lung cancer, MPN: malignant pulmonary nodule, BPN: benign pulmonary nodule, *:*P*<0.05,**:*P*<0.01,***:*P*<0.001

As shown in Fig. 2a, the level of anti-UBQLN1 in LC was obviously higher than that in NC (*P*<0.05) as well as in BLD (*P=*0.019). The AUC of anti-UBQLN1 was 0.608 and 0.574 in discriminating LC from NC or BLD, with the sensitivity of 19.3% or 18.4% and the specificity of 93.9% or 92.4%, respectively (Fig. 2b, c). The analysis of anti-UBQLN1 in different subgroup of validation cohort 1 indicated that it existed significant differences between early LC and advanced LC, NSCLC and SCLC, male and female LC patients. (*P*<0.05, Fig. 2d).

To further explore the accuracy of ELISA, we validated the titer of anti-UBQLN1 in 11 LC samples and 11 NC samples via western blotting (WB). Figure S2 displayed that 8 of eleven LC samples showed positive bands while 1 of twelve NC samples showed positive bands with approximately 70 KD.

### Combination of anti-UBQLN1, traditional serum biomarkers and CT indicators can improve the discrimination accuracy of PNs

**Fig. 3:**
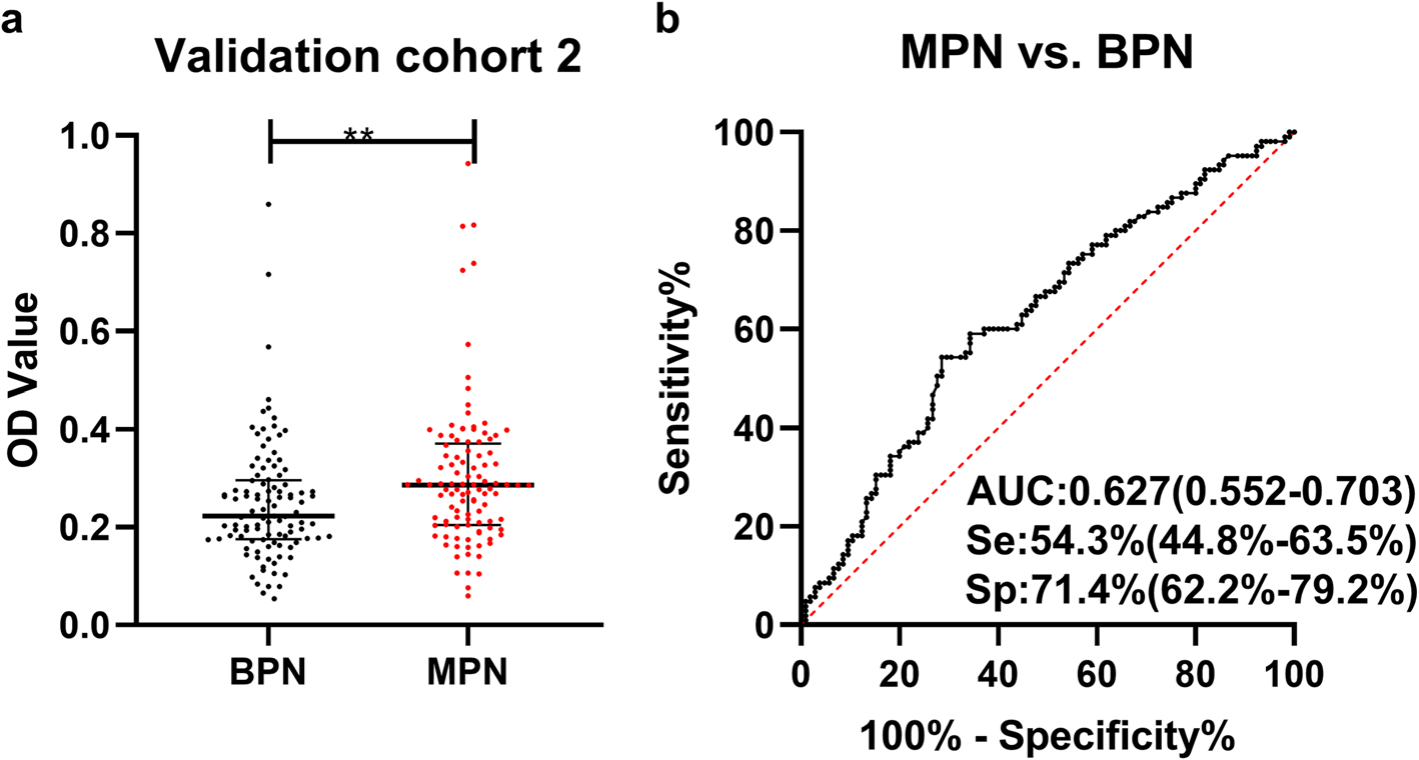
The diagnostic ability of anti-UBQLN1 in validation cohort 2. a. scatter plot of OD value of anti-UBQLN1 in validation cohort 2. b. ROC of anti-UBQLN1 for the discrimination between MPN and BPN patients.

Figure 3a illustrated the level of anti-UBQLN1 in validation cohort 2. Anti-UBQLN1 can discriminate BPN from MPN patients with the AUC (*95%CI*) of 0.627 (0.552-0.703) (Fig. 3b).

The 12 CT nodular characteristics in 210 PN patients of validation cohort 2 were displayed in Table 2. 10 of 12 CT indicators showed a significant difference between MPN patients and BPN patients (*P*<0.05). In validation cohort 2, anti-UBQLN1, 3 traditional biomarkers (CEA, CYFRA211 and CA125) and 10 nodular characteristics of CT (number, diameter, cavity, spicule sign, vascular notch sign, lobulation sign, spines, pleural indentation, mediastinal lymph node enlargement and calcification) were employed to construct model for the differentiation of PN patients based on logistic regression analysis.

**Table 2:** Nodular characteristic of CT in validation cohort 2 BPN benign pulmonary nodule, MPN malignant pulmonary nodule, P X ^2^ test

*Nodular Characteristic of CT*	MPN (%) (*n*=105)	BPN (%) (*n*=105)	*P*
*Number*			<0.001^*^
1	87(82.9)	60(57.1)	
>1	18(17.1)	45(42.9)	
*Diameter(mm)*			
Mean±SD	28.58±19.90	20.00±16.14	0.002*
Range	5.3-130.9	2.0-76.5	
*Edge sign*			0.072
Yes	26(24.8)	38(36.2)	
No	79(75.2)	67(63.8)	
*Empty sign*			0.023^*^
Yes	8(7.6)	19(18.1)	
No	97(92.4)	86(81.9)	
*Spicule sign*			0.004^*^
Yes	30(28.6)	13(12.4)	
No	75(71.4)	92(87.6)	
*Vascular notch sign*			<0.001^*^
Yes	80(76.2)	48(45.7)	
No	25(23.8)	57(54.3)	
*Lobulation sign*			<0.001^*^
Yes	55(52.4)	13(12.4)	
No	50(47.6)	92(87.6)	
*Spines*			0.009^*^
Yes	18(17.6)	6(5.7)	
No	87(82.9)	99(94.3)	
*Pleural indentation*			0.004^*^
Yes	35(33.3)	17(16.2)	
No	70(66.7)	88(83.8)	
*Mediastinal lymph node enlargement*			0.003^*^
Yes	36(34.3)	17(16.2)	
No	69(65.7)	88(83.8)	
*Emphysema*			0.408
Yes	21(20.0)	26(24.8)	
No	84(80.0)	79(75.2)	
*Calcification*			<0.001^*^
Yes	3(2.9)	25(23.8)	
No	102(97.1)	80(76.2)	

**Fig. 4:**
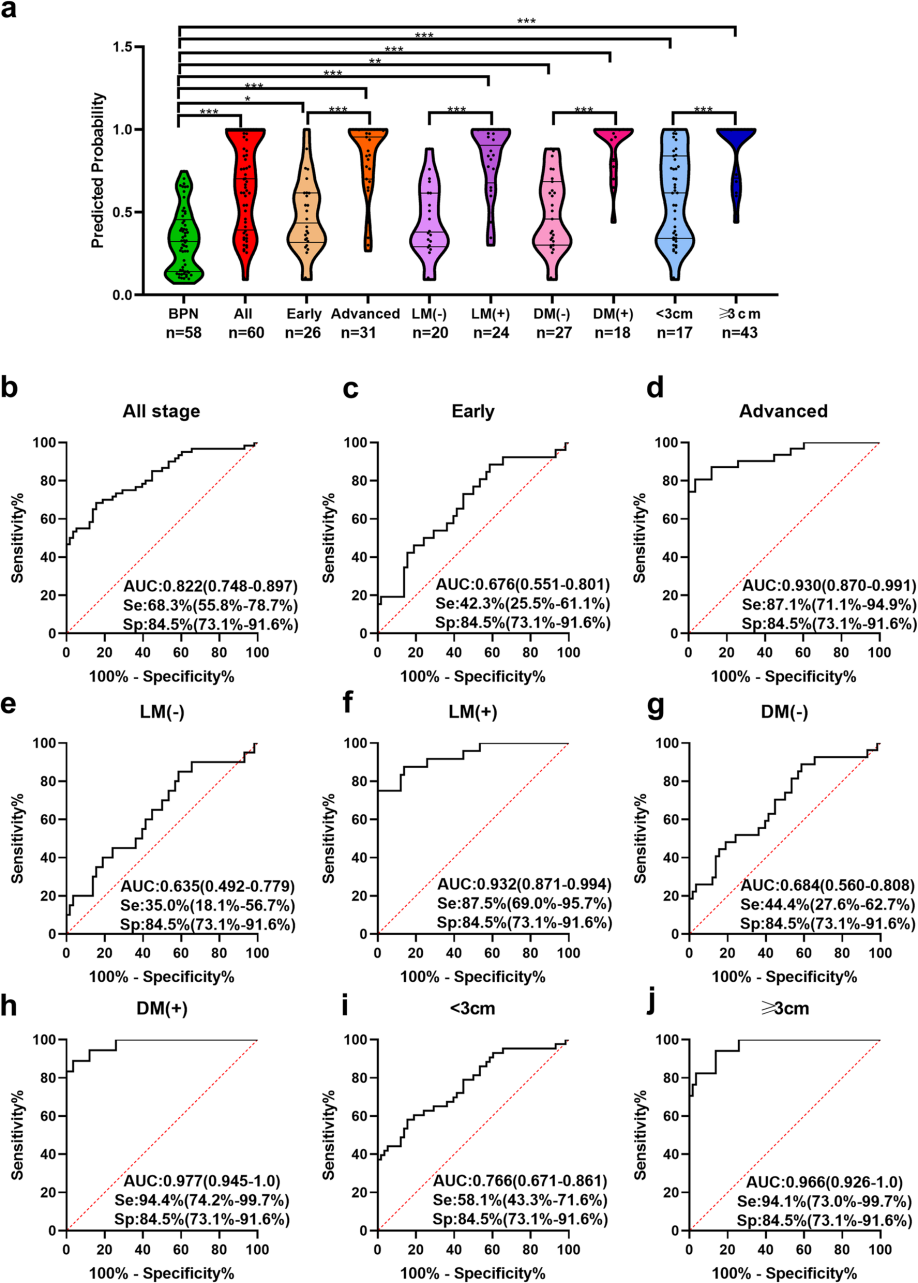
Evaluation of the diagnostic model in validation cohort 2. a: The violin plot and scatter plot of predicted probability of model in MPN patients with different clinical character and BPN patients. b-j: ROC of model for distinguishing MPN patients with different clinical character from BPN. n: number, ALL: all MPN, Early: early stage MPN, Advanced: advanced stage MPN, LM(-): Lymph node metastasis negative, LM(+): Lymph node metastasis positive, DM(-): distant metastasis negative, DM(+): distant metastasis positive, <3cm: diameter<3cm, ≥3cm: diameter ≥3cm, *: *P*<0.05, **: *P*<0.01, ***: *P*<0.001, Lines represented median and quartile range

One hundred eighteen individuals (60 MPN patients and 58 BPN patients) with both of the result of traditional biomarkers and CT were selected for the further research. A model consisted of anti-UBQLN1, traditional biomarkers (CEA, CYFRA211 and CA125) and 3 nodular characteristics of CT (vascular notch sign, lobulation sign and mediastinal lymph node enlargement) with the AUC of 0.822 (*95%CI*:0.748-0.897) were showed in Fig. 4b, which dramatically improve the diagnostic ability of single diagnostic approach (Fig. S3). The predicted possibility for discrimination as MPN was $\mathrm{PRE}\;=\;1/(1+\mathrm{EXP}(-(+2.629\times\mathrm{anti}-\mathrm{UBQLN}1+1.139\times\mathrm{vascular}\;\mathrm{notch}\;\mathrm{sign}\;+1.117\times\mathrm{lobulation}\;\mathrm{sign}\;+0.794\times\mathrm{mediastinal}\;\mathrm{lymph}\;\mathrm{node}\;\mathrm{enlargement}\;+\;0.155\times\mathrm{CEA}\;+\;0.001\times\mathrm{CA}125\;+\;0.188\times\mathrm{CYFRA}211-3.161)))$.

Sixty MPN patients were stratified by the clinical characteristics of tumor stage, nodular diameter, LM and DM. The discriminating performance of the model for MPN patients in the different characteristics was described in Fig. 4 and Table 3. The model owned the highest AUC of 0.977 in differentiating positive distant metastatic (DM+) MPN from BPN patients (Fig. 4h) while it exhibited the lowest AUC of 0.676 in the discrimination of early MPN patients (Fig. 4c). Besides, it exhibited a better discrimination performance in patients with advanced, positive lymph node metastatic (LM+), DM+ and diameter ≥3 cm MPN (Fig. 4d, f, h, j) in comparison with patients with early, negative lymph node metastatic (LM-), negative distant metastatic (DM-) and diameter <3 cm MPN (Fig. 4c, e, g, i).

**Table 3:** Diagnostic performance of model in MPN patients with different clinical characteristic

Group	n	Se (%)	Sp (%)	+LR	-LR	PPV (%)	NPV (%)	YI	Accuracy (%)
All stage MPN vs. BPN	60	68.3	84.5	4.406	0.375	0.82	0.721	0.528	76.3
Early MPN vs. BPN	26	42.3	84.5	2.729	0.682	0.55	0.766	0.268	71.4
Advanced MPN vs. BPN	31	87.1	84.5	5.619	0.153	0.75	0.924	0.716	85.4
LM(-) MPN vs. BPN	20	35.0	84.5	2.258	0.769	0.44	0.790	0.195	71.8
LM(+) MPN vs. BPN	24	87.5	84.5	5.645	0.148	0.70	0.942	0.720	85.4
DM(-) MPN vs. BPN	27	44.4	84.5	2.865	0.658	0.57	0.766	0.289	71.7
DM(+) MPN vs. BPN	18	94.4	84.5	6.091	0.066	0.65	0.980	0.789	86.8
≥3cm MPN vs. BPN	43	94.1	84.5	6.071	0.070	0.82	0.942	0.786	88.1
<3cm MPN vs. BPN	17	58.1	84.5	3.748	0.496	0.52	0.875	0.426	78.7

*MPN* malignant pulmonary nodule, *BPN* benign pulmonary nodule, *Se* sensitivity, *Sp* specificity, *AUC* area under curve, 95%*CI* 95% confidence interval, +*LR* positive likelihood ratio, -*LR* negative likelihood ratio, *PPV* positive predictive value, *NPV* negative predictive value, *YI* Youden’s index, *Advanced* advanced LC, *LM*(-) Lymph node metastasis negative, *LM*(+) Lymph node metastasis positive, *DM*(-) distant metastasis negative, *DM*(+) distant metastasis positive, *<3cm* diameter<3cm, *≥3cm* diameter≥3cm

### UBQLN1 protein was overexpressed in adenocarcinoma tissues in comparison with para-tumor lung tissues

**Fig. 5:**
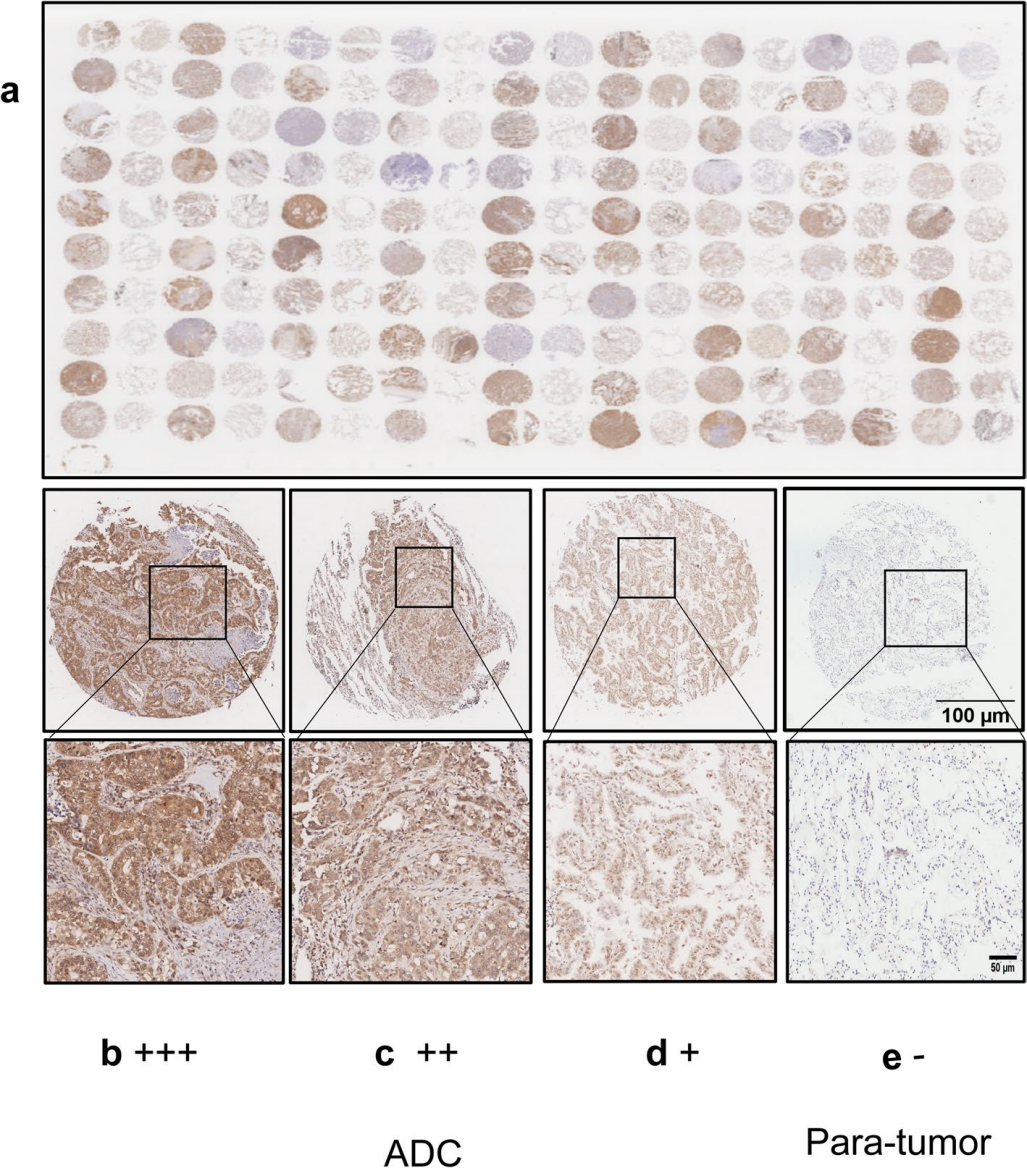
Expression of UBQLN1 in ADC tissues and para-tumor tissues a. The expression of UBQLN1 protein in 88 para-tumor tissues and 88 ADC tissues; b. ADC: Strong positive expression of UBQLN1 protein at ×30 (scale bar:100 μm) and ×90 magnification (scale bar:50 μm). c. ADC: Medium positive expression of UBQLN1 protein at ×30 (scale bar:100 μm) and ×90 magnification (scale bar:50 μm). d. ADC: Weak positive expression of UBQLN1 protein at at ×30 (scale bar:100 μm) and ×90 magnification (scale bar:50 μm). e. Para-tumor: negative expression of UBQLN1 protein at at ×30 (scale bar:100 μm) and ×90 magnification (scale bar:50 μm).

**Fig. 6:**
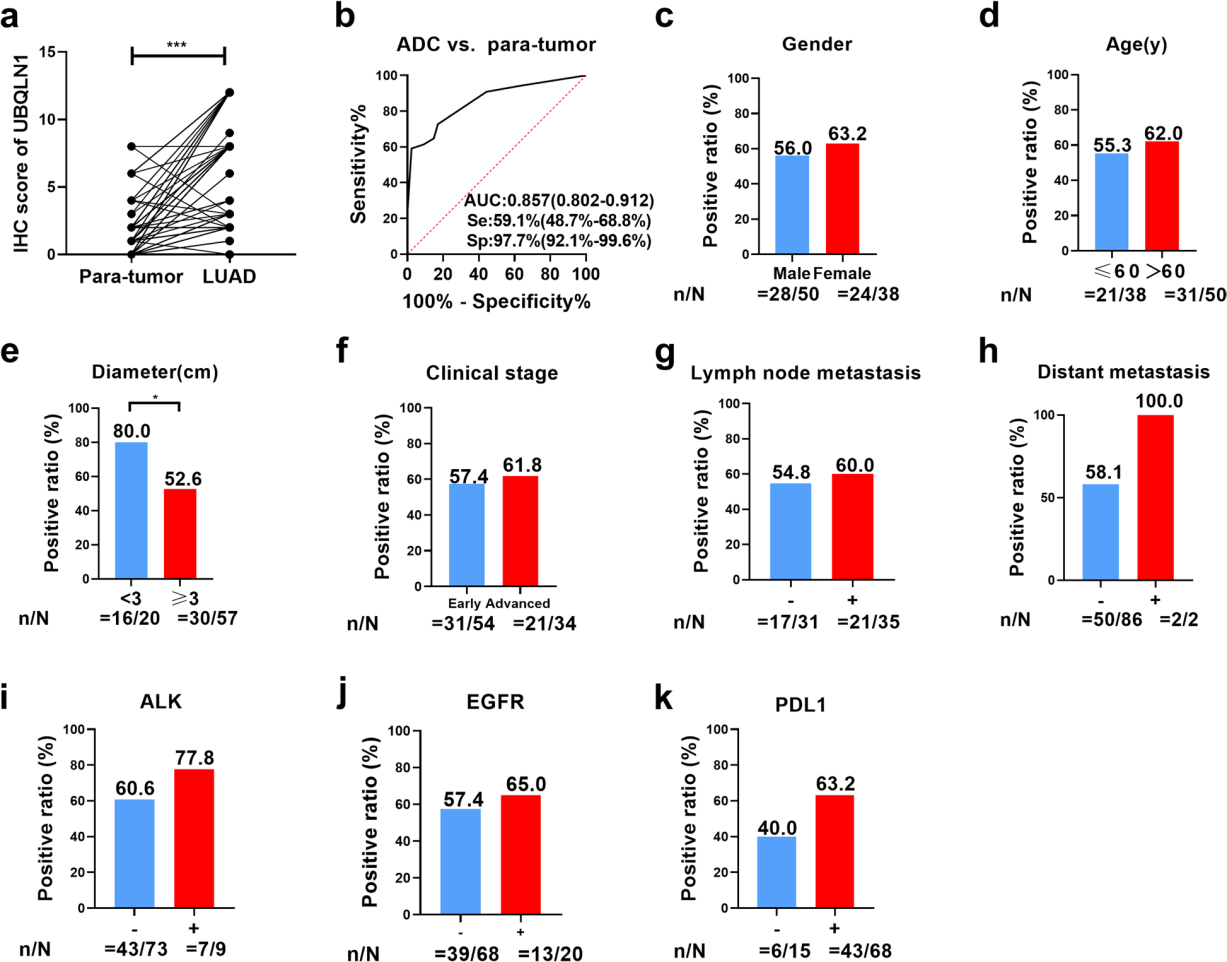
Analysis of UBQLN1 expression in 88 ADC and 88 para-tumor tissues. a. The analysis of UBQLN1 protein expression in ADC tissues and the corresponding normal tissues b. ROC analysis of UBQLN1 protein in ADC tissues and the corresponding tissues. c-k. Comparison of positive ratio of UBQLN1 expression in different gender, age, diameter, clinical stage, lymph node metastasis, distant metastasis, ALK expression, EGFR expression and PDL1 expression. *: *P*<0.05, **: *P*<0.01, ***: *P*<0.001, Lines represented median and quartile range

Figure 5 displayed the representative images of IHC results. The detailed information about patients from tissue array was described in Table S1. The total score in LC tissues was significantly higher than that in adjacent tissues (*P*<0.05, Fig. 6a). Furthermore, the AUC of UBQLN1 protein is 0.867 in differentiating ADC from para-tumor tissues (Fig. 6b). According to total score of IHC, the expression of UBQLN1 protein was divided into two groups: high expression and low expression group. Additionally, there are no obvious differences of positive ratio among gender, age, clinical stage, lymph node metastasis, distant metastasis, ALK mutation status, EGFR mutation status and PDL1 expression (*P*>0.05) (Fig. 6c-d, f-k) except diameter (Fig. 6e, Table 4) between high UBQNL1 expression and low UBQNL1 expression.

**Table 4:** Relation of UBQLN1 expression and different clinicopathologic characteristic

	UBQLN1		P
	High expression (%)	Low expression (%)	
*Gender*			
male	28(56.0)	22(44.0)	0.499
female	24(63.2)	14(36.8)	
*Age(y)*			
≤60	21(55.3)	17(44.3)	0.524
>60	31(62.0)	19(38.0)	
*Diameter(cm)*			
<3	16(80.0)	4(20.0)	0.032*
≥3	30(52.6)	27(47.4)	
*Clinical stage*			
Early	31(57.4)	23(42.6)	0.686
Advanced	21(61.8)	13(38.2)	
*LM*			
Yes	21(60.0)	14(40.0)	0.672
NO	17(54.8)	14(45.2)	
*DM*			
Yes	2(100.0)	0(0.0)	0.511
No	50(58.1)	36(41.9)	
*ALK expression*			
Yes	7(77.8)	2(22.2)	0.471
No	43(60.6)	28(39.4)	
*EGFR mutation*			
Yes	13(65.0)	7(35.0)	0.541
No	39(57.4)	29(42.6)	
*PDL1 expression*			
Yes	43(63.2)	25(36.8)	0.098
No	6(40.0)	9(60.0)	

Early: early LC, Advanced: advanced LC, *LM* Lymph node metastasis, *DM* distant metastasis

^*^There are significant difference among different groups

### Knockdown of UBQLN1 could inhibit the migration, invasion and proliferation of LC cell lines

**Fig. 7:**
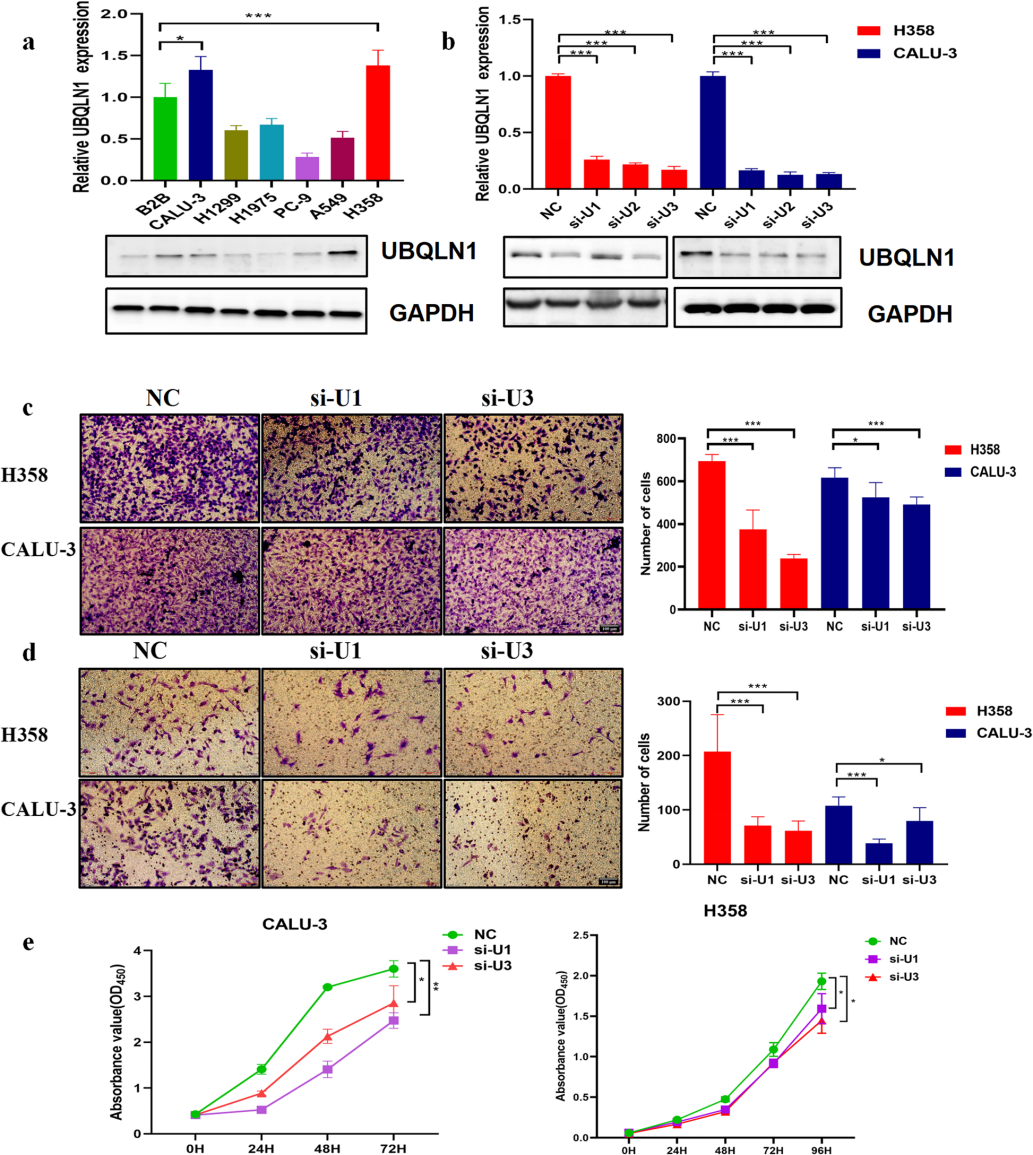
Profiles of representative images about UBQLN1 and the role of UBQLN1 in cell invasion, migration and proliferation of lung cancer. UBQLN1 expression of a normal lung cell line (B2B) compared with NSCLC cell lines (CALU-3, PC-9, H1975, H1299, A549 and H358) was measured by qRT-PCR (bar plot, upper panel) and western blotting (Cropped strips). UBQLN1 level of two cell lines (H358 and CALU-3) with UBQLN1 knockdown (si-U1, si-U2, si-U3) in comparison with NC was measured by cell western blotting (Cropped strips) and qRT-PCR. c-d. Knockdown of UBQLN1 could decrease the ability of invasion (c) and migration (d) in two cell lines (H358 and CALU-3), magnification: ×100, scale bar: 100 μm. e. Knockdown of UBQLN1 could decrease the ability of proliferation in two cell lines (H358 and CALU-3). NC: siNC, si-U1: si-UBQLN1-1, si-U2: si-UBQLN1-2, si-U3: si-UBQLN1-3*:*P*<0.05,**:*P*<0.01,***:*P*<0.001

We selected 7 cell lines in order to detect the expression of UBQLN1 protein in LUAD and normal cell lines (B2B). Of the 6 LUAD cell lines, the expression of UBQLN1 protein in two cell lines (H358 and CALU-3) was obviously higher than that in B2B whereas its expression in other four cell lines (H1299, H1925, PC-9 and A549) was lower than that in B2B at the mRNA and protein level (Fig. 7a). Therefore, H358 and CALU-3 cell lines which overexpressed UBQLN1 protein were selected for the further research.

Fig. 7b indicated that three siRNA can significantly reduce the expression of UBQLN1 mRNA and protein in these two cell lines in comparison with negative control. We choose H358 and CALU-3 cell lines transfected si-U1 and si-U3 to investigate its function in cell migration, invasion and proliferation. Silencing UBQLN1 remarkably inhibited the migration, invasion and proliferation of two cell lines (Fig. 7c-e).

## Discussions

To date, many researchers still focused on the identification of potential biomarkers with excellent diagnostic performance which can be applied in the clinic so that they can screen high risk individuals at an early stage [8, 10, 11, 21, 22]. Hence, appropriate treatment can be implemented to further reduce the morality rate and improve the survival rate of advanced patients.

In a study conducted by Chen et al, anti-UBQLN1 was previously proved that it can differentiate lung ADC patients from normal controls with the AUC of 0.84 [16]. Furthermore, another study has demonstrated that it also can distinguish 6% of ovarian cancer (OC) patients (3 out of 50 OC patients) [23]. In the current study, we aim to explore the diagnostic capability of anti-UBQLN1 for the detection of LC. Additionally, we also examined the level of autoantibodies to UBQLN1 in BLD patients. It can be seen from our study that anti-UBQLN1 can discriminate LC from BLD patients. It is the first research to investigate the discrimination ability of anti-UBQLN1 for the discrimination of PN with the AUC of 0.610. In our research, we confirmed that anti-UBQLN1 could be a potential serum biomarker of LC.

As our previous study has proved that it is very common to screen single biomarker with low sensitivity [19], the merit of anti-UBQLN1 is that it not only can diagnose high-risk lung cancer patients from healthy individuals but also can be a promising biomarker in the discrimination of pulmonary nodules. Another possible reason attributed to low AUC of anti-UBQLN1 is that only 10 paired serum was used to screen the candidate biomarker through protein microarray, resulting in the lack of convincing evidence for the diagnosis capability of anti-UBQLN1. Most of the previous studies showed that combining multiple biomarkers can remarkably improve the diagnostic accuracy and specificity in different cancerous diseases, such as OC, BC, ESCC, gastric cancer (GC) and LC [17, 24–28]. Jiang et al. utilized decision tree method to construct a diagnostic panel consists of seven TAAbs (TP53, NPM1, FGFR2, PIK3CA, GNA11, HIST1H3B, and TSC1) with the AUC of 0.897 and sensitivity and specificity of 94.4% and 84.9% [17]. Pei et al. applied logistic regression to build a model for the diagnosis of LC patients [10]. Hence, we employed logistic regression to combine it with other traditional biomarkers and CT indicators for improving the diagnostic performance of single biomarker in our study. The AUC of this model was up to 0.822 (0.748-0.824) with the sensitivity and specificity of 68.3% and 84.5%. The result showed that the combination of several biomarker and CT indicators can be an effective method to improve the diagnostic ability of diseases.

Moreover, previous researches showed that the expression of UQBLN1 protein was thought to be a prognostic marker for the development of diseases [29, 30]. Xu et al. showed that overexpression of UBQLN1 in most cases implied the poor prognosis of hepatocellular carcinoma [31].Wang et al. discovered that the overexpression of UBQLN1 was found in breast cancer (BC) tissue, which was associated with tumor size, lymph node metastasis, TNM stage, vascular invasion and poor prognosis of BC patients [30]. Bao et al. discovered that UBQLN1 was obviously upregulated in gastric cancer and related to worse prognosis of GC patients [29]. Therefore, we tried to investigate the relationship between the level of UBQLN1 and the prognosis of LC patients. In the present study, we found that the level of UBQLN1 protein was obviously higher in lung ADC tissue in comparison with para-cancerous tissues. Only tumor diameter was significantly correlated with UBQLN1 expression (*P*<0.05). The relationship between UBQLN1 expression and other clinical characteristics (gender, age, clinical stage, lymph node metastasis, distant metastasis, ALK expression, EGFR expression and PDL1 expression) was not found. It might be attributed to small sample size and sample type. Later, our further researches aim to collect more samples to discover the relation between UBQLN1 expression and other clinical characteristics. Furthermore, no significant difference was discovered between the survival time of patients and UBQLN1 expression (*P*>0.05). This result showed that UBQLN1 expression was not correlated with LC prognosis. Several previous studies demonstrated that UBQLN1 was involved in crucial biological processes of several cancers [14, 31–36], especially epithelial-to-mesenchymal transition (EMT). EMT is a process in which epithelial cells acquire mesenchymal features. In cancer, EMT is associated with tumor initiation, invasion, metastasis, and resistance to therapy [37]. Many epithelial and mesenchymal markers such as E-cadherin and vimentin had been proved to be a significant biomarker for discriminating EMT status. Moreover, EMT plasticity is an important factor in immune escape and therapy resistance such as EGFR-targeted therapy in lung cancer [38]. EGFR-Tyrosine Kinase Inhibitors (EGFR-TKIs) is a common method used for the treatment of LC patients. Many experimental studies and case reports documenting EMT in EGFR-TKI-resistant NSCLC investigated also genetic alterations, which commonly occur in relapsed tumors and activate alternative pathways bypassing EGFR addiction. In addition, EMT may override the immunosuppression evolving in EGFR-TKI-resistant tumors and targeting the EMT state may improve the response to treatments combining EGFR-TKIs with the immunotherapy [38]. Hence, it is of importance to understand the relationship between UBQLN1 and EMT pathways in different kinds of cancers. Feng et al. discovered that UBQLN1 silencing can inhibit EMT and MMP expression via AKT signaling in breast cancer [36]. Shah et al. found that UBQLN1 played an important part in EMT of human non-small cell lung cancer cells and repressed migration [14]. One study published in 2022 proved that loss of UBQLN1 induces tumor progression and metastasis, including proliferation, clonogenic potential and migration in A549 cell lines [39]. In our study, the function experiments also proved that knockdown of UBQLN1 can inhibit the proliferation of LUAD cell lines (H358 and CALU3). However, loss of UBQLN1 can inhibit the invasion and migration that differ from the former conclusion. Moreover, this study mainly focused on the value of UBQLN1 and anti-UBQLN1 as a diagnostic and prognostic biomarker and we failed to validate the function of UBQLN1 in-vivo experiment and its effect in the related pathways such as EMT, PIK3 and AKT pathway. In the further research, more experiments would be conducted to explore the interaction between UBQLN1 and EMT or other pathways. Moreover, UBQLN1 is proved to be a resistance-related factor and a novel target for therapeutic. One previous study showed that knockdown of UBQLN1 enable resistant cells to resensitize to sorafeni and provide a potential signaling pathway and novel targets for combination therapies [40]. However, little research is related to the resistance of lung cancer by targeting UBQLN1 until now. Hence, we would pay more attention to drug-resistance lung cancer and find more effective target for achieving higher treatment efficiency.

### Supplementary Information

**Supplementary Material 1.**

**Supplementary Material 2.**

**Supplementary Material 3.**

**Supplementary Material 4.**

**Supplementary Material 5.**

**Supplementary Material 6.**

**Supplementary Material 7.**

**Supplementary Material 8.**

## Data Availability

The datasets used and/or analyzed during the current study are available from the corresponding author on reasonable request.

## Abbreviations

LC: lung cancer
NC: normal control
BLD: benign lung disease
PN: pulmonary nodule
MPN: malignant pulmonary nodule
BPN: benign pulmonary nodule
UBQLN1: Ubiquilin 1
anti-UBQLN1: autoantibody to UBQLN1
ELISA: Enzyme-linked immunosorbent assay
ROC: Receiver operating characteristic curve
CCK-8: cell counting kit-8
AUC: The area under the curve
LUAD: lung adenocarcinoma
ADC: lung adenocarcinoma
SCC: squamous cell carcinoma
NSCLC: non-small cell lung cancer
SCLC: small cell lung cancer
COPD: chronic obstructive pulmonary disease
CB: chronic bronchitis
LDCT: low-dose computed tomography
TAAs: tumor associated antigens
TAAbs: autoantibodies against TAAs
SEREX: serological analysis of recombination cDNA expression libraries
SERAP: serological proteome analysis
WB: western blotting
SD: standard deviation
BCA: Bicinchoninic acid
SDS-PAGE: polyacrylamide gelelectrophoresis
PVDF: polyvinylidene fluoride
HRP: horseradish peroxidase
ECL: electrochemiluminescence
qRT-PCR: Quantitative RT-PCR
OD: optional density
IHC: immunohistochemistry
YI: Youden’s index
FC: fold change
DM+: positive distant metastatic
DM-: negative distant metastatic
LM+: positive lymph node metastatic
LM-: negative lymph node metastatic
BC: breast cancer
GC: gastric cancer
EMT: epithelial-to-mesenchymal transition
EGFR-TKIs: EGFR-Tyrosine Kinase Inhibitors
