# Supplementary material for: Humoral immune response to tumor-associated antigen Ubiquilin 1 (UBQLN1) and its tumor-promoting potential in lung cancer
Source: BMC Cancer. 2024 Mar 2;24:283. doi: 10.1186/s12885-024-12019-w (PMC10908023; doi:10.1186/s12885-024-12019-w)
Supplement: Supplementary file 1 — Supplementary Material 1. [file 12885_2024_12019_MOESM1_ESM.docx]

**Figure S1**. **Results of UBQLN1 based on Huprot protein microarray**


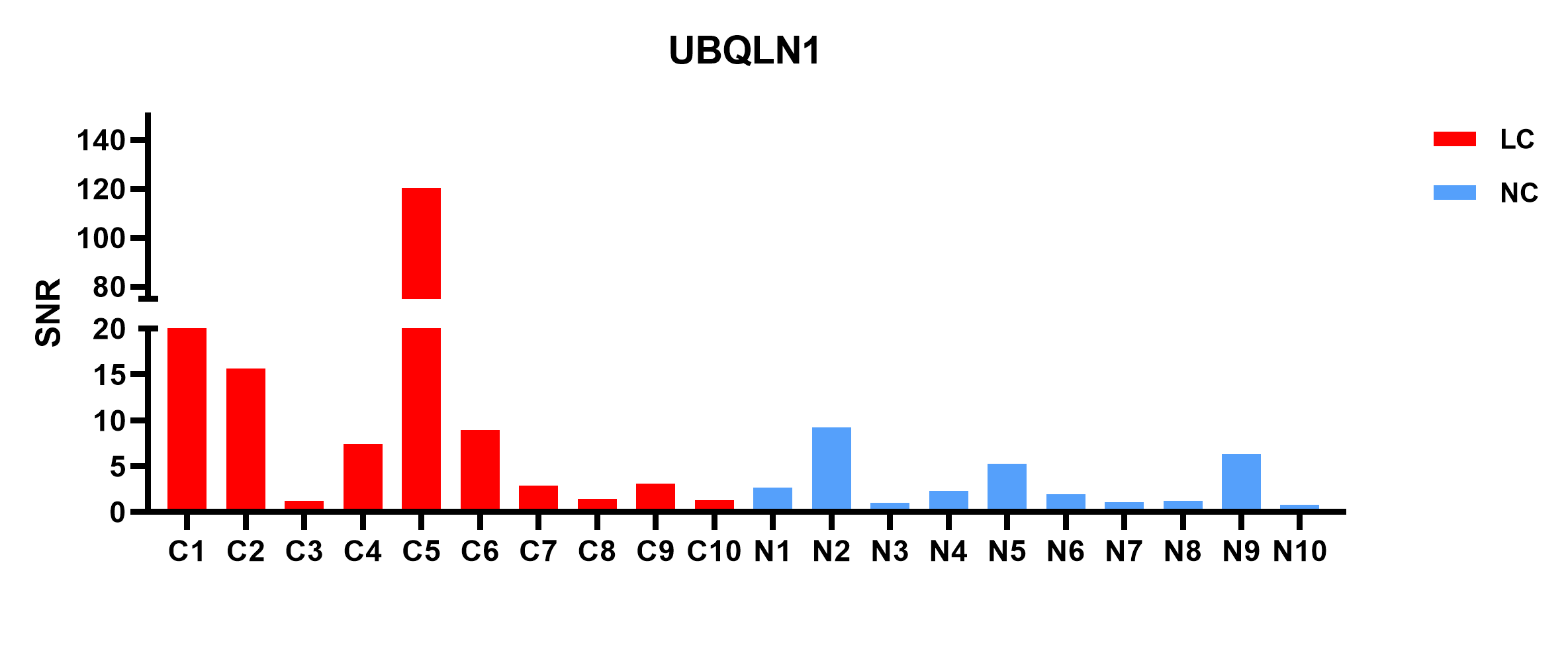


LC: lung cancer, NC: normal control

**Figure S2. Representative cropping strips of** **anti-UBQLN1 in sera from 11 LC patients and 11 NC** **by western blotting.**


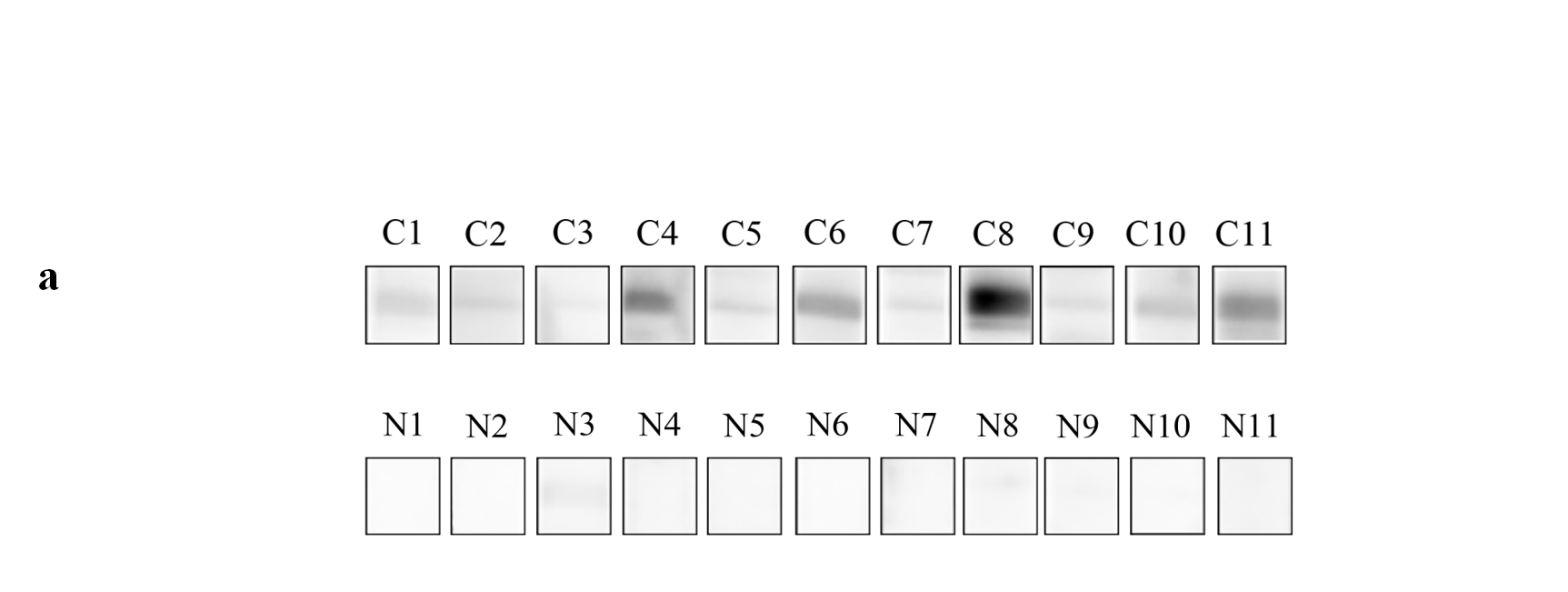


C: lung cancer, N: normal control

**Figure S3. The diagnostic ability of single CT indicator and serum biomarker.**


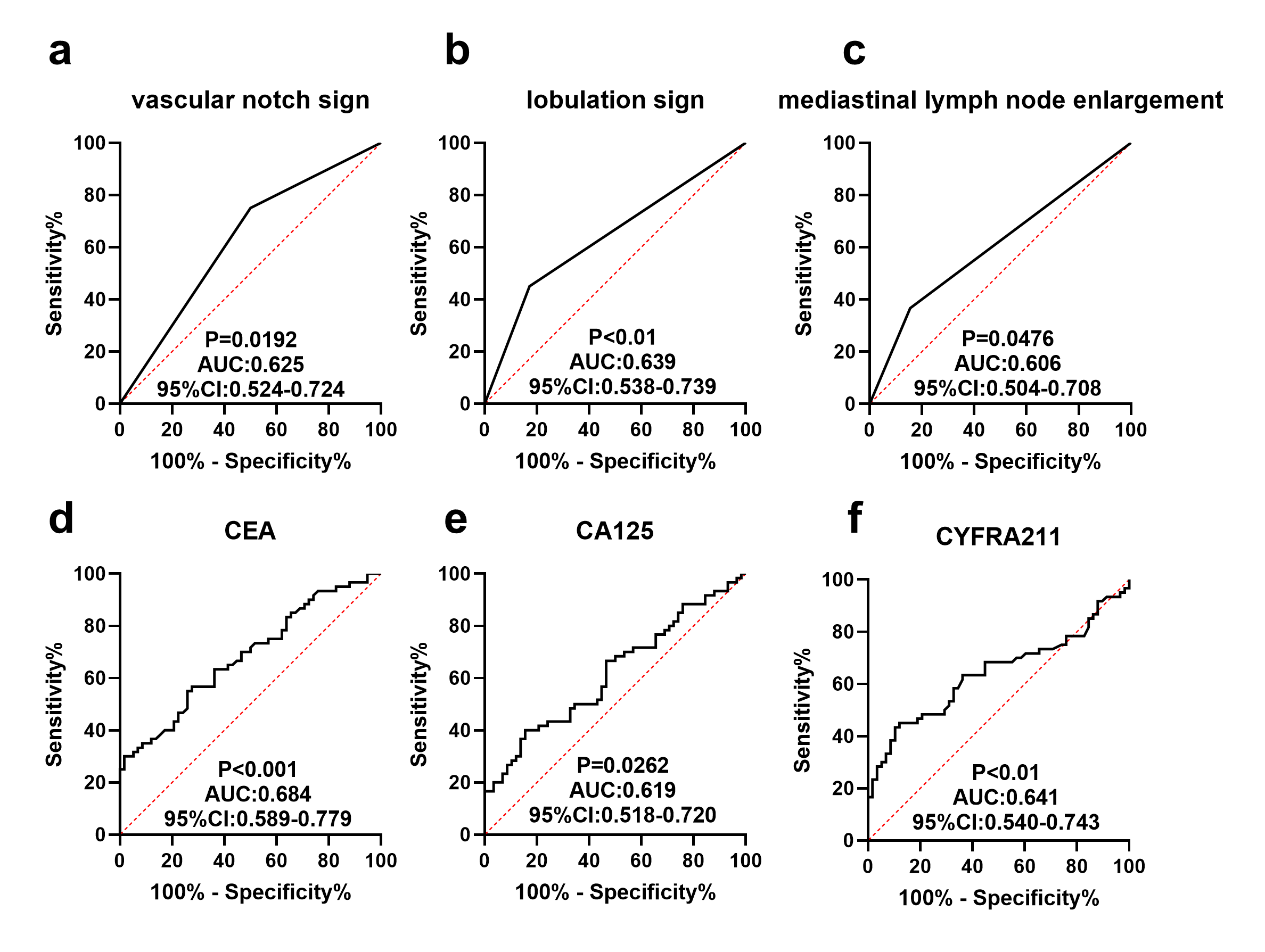


a-f. ROC of vascular notch sign, lobulation sign, mediastinal lymph node enlargement, CEA, CA125 and CYFRA211.

**Table S1. The clinical characteristic of ADC tissues and adjacent tissues**

|  | **ADC(%)** | **adjacent tissues (%)** |
| --- | --- | --- |
| ***N*** | 88 | 88 |
| ***Gender*** |  |  |
| Female | 41（46.6） | 41（46.6） |
| Male | 47（53.4） | 47（53.4） |
| ***Age（y）*** |  |  |
| ＜60 | 33（37.5） | 33（37.5） |
| ≥60 | 55（62.5） | 55（62.5） |
| ***Clinical stage*** |  |  |
| I | 0（0.0） |  |
| II | 54（61.4） |  |
| III | 34（38.6） |  |
| IV | 0（0.0） |  |
| ***Lymph node Metastasis*** |  |  |
| Yes | 35（39.8） |  |
| No | 31（35.2） |  |
| Unknown | 22(25.0) |  |
| ***Distant metastasis*** |  |  |
| Yes | 2（2.3） |  |
| No | 86（97.7） |  |
| ***ALK mutation*** |  |  |
| Yes | 71（80.7） |  |
| No | 9（10.2） |  |
| Unknown | 8（9.1） |  |
| ***PDL1 expression*** |  |  |
| Yes | 68（77.3） |  |
| No | 15（17.0） |  |
| Unknown | 5（5.7） |  |
| ***EGFR mutation*** |  |  |
| Yes | 20（22.7） |  |
| No | 68（77.3） |  |
