# Supplementary figures and images for: Humoral immune response to tumor-associated antigen Ubiquilin 1 (UBQLN1) and its tumor-promoting potential in lung cancer
Source: BMC Cancer. 2024 Mar 2;24:283. doi: 10.1186/s12885-024-12019-w (PMC10908023; doi:10.1186/s12885-024-12019-w)

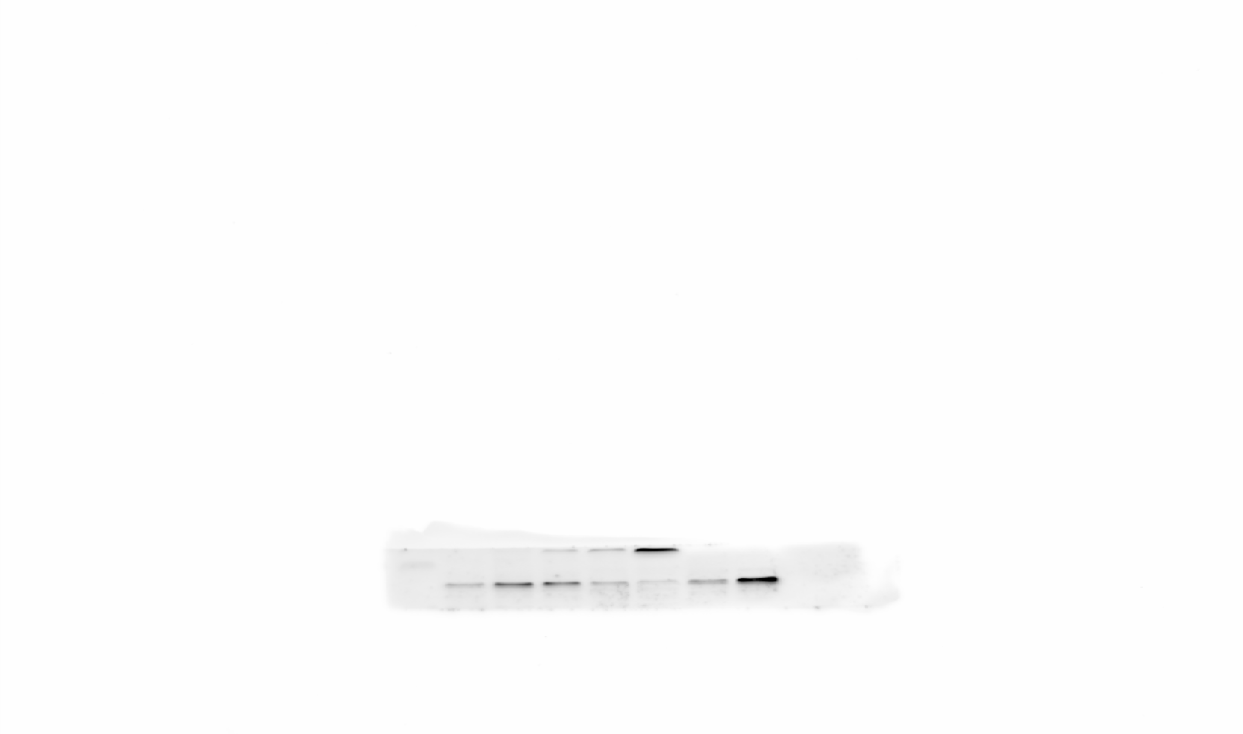

Supplement: Supplementary file 2 — Supplementary Material 2. [file 12885_2024_12019_MOESM2_ESM.png]

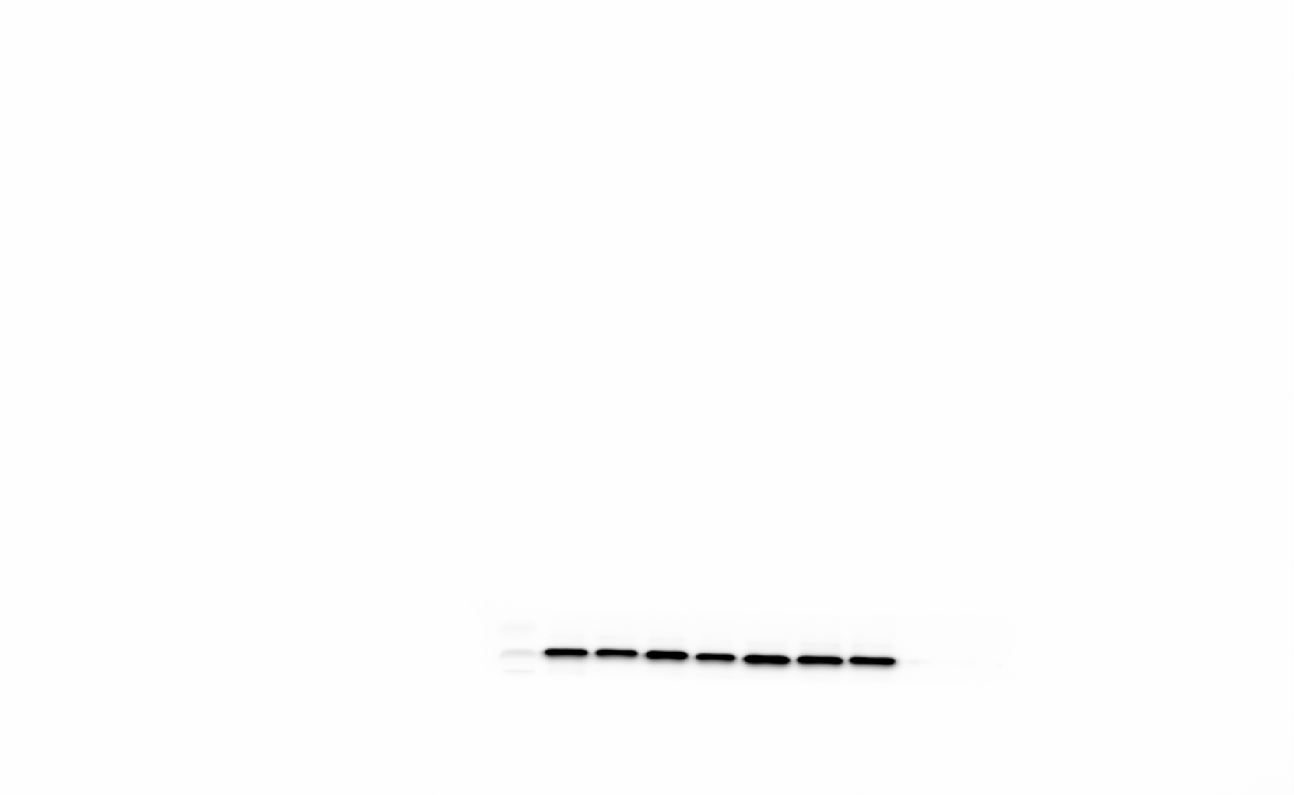

Supplement: Supplementary file 3 — Supplementary Material 3. [file 12885_2024_12019_MOESM3_ESM.png]

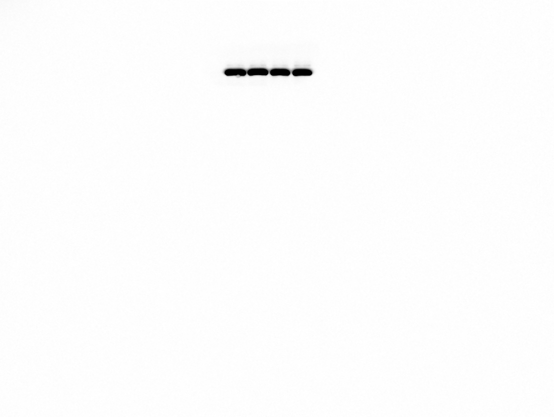

Supplement: Supplementary file 4 — Supplementary Material 4. [file 12885_2024_12019_MOESM4_ESM.png]

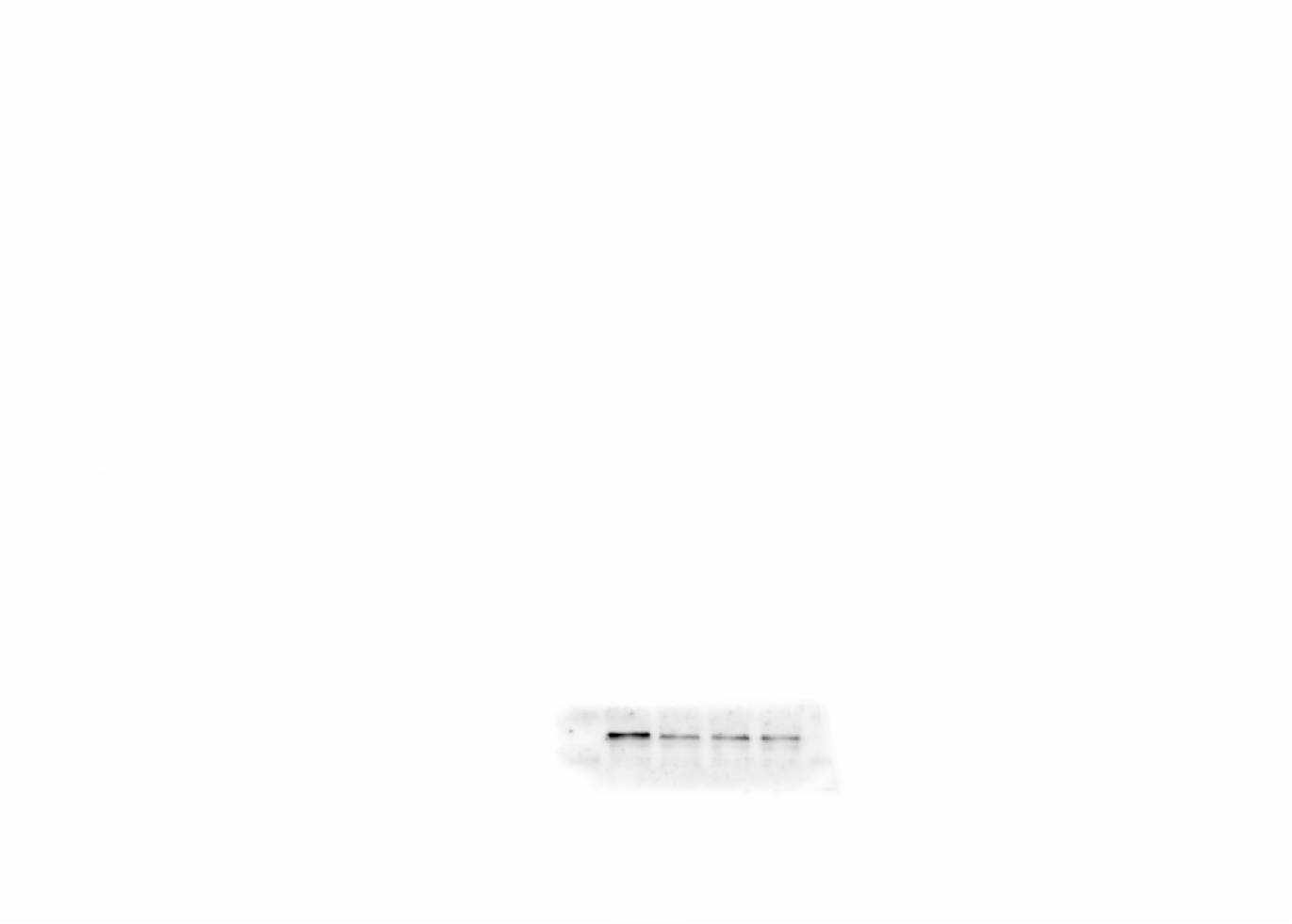

Supplement: Supplementary file 5 — Supplementary Material 5. [file 12885_2024_12019_MOESM5_ESM.png]

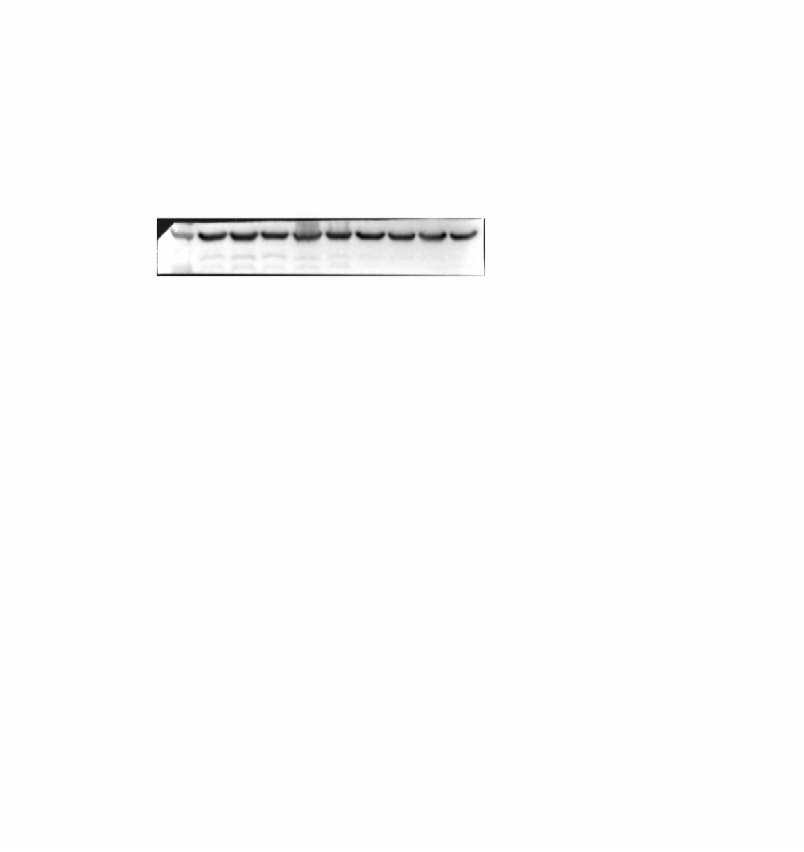

Supplement: Supplementary file 6 — Supplementary Material 6. [file 12885_2024_12019_MOESM6_ESM.png]

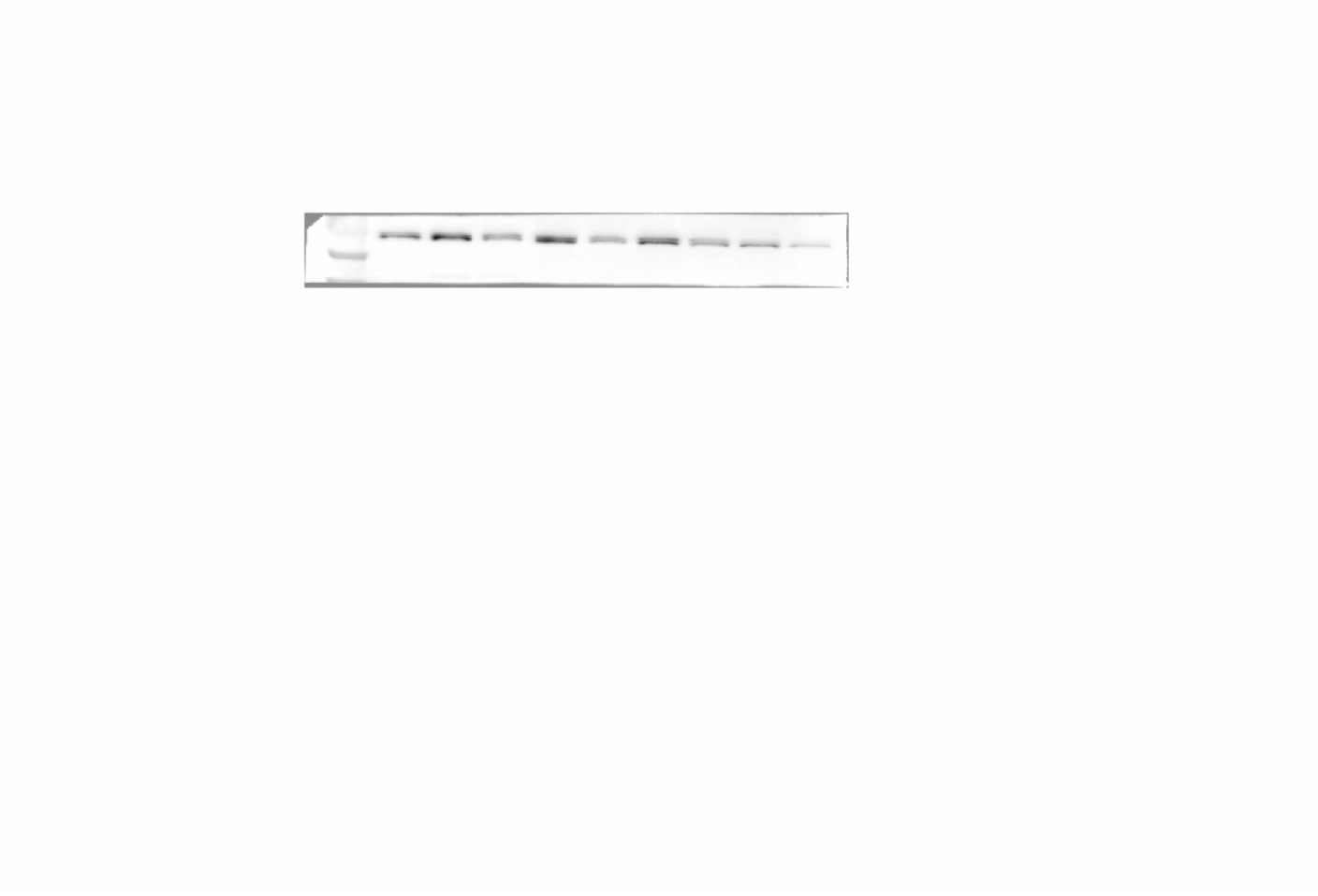

Supplement: Supplementary file 7 — Supplementary Material 7. [file 12885_2024_12019_MOESM7_ESM.png]
