# Supplementary material for: Humoral immune response to tumor-associated antigen Ubiquilin 1 (UBQLN1) and its tumor-promoting potential in lung cancer
Source: BMC Cancer. 2024 Mar 2;24:283. doi: 10.1186/s12885-024-12019-w (PMC10908023; doi:10.1186/s12885-024-12019-w)

Fig7a-UBQLN1

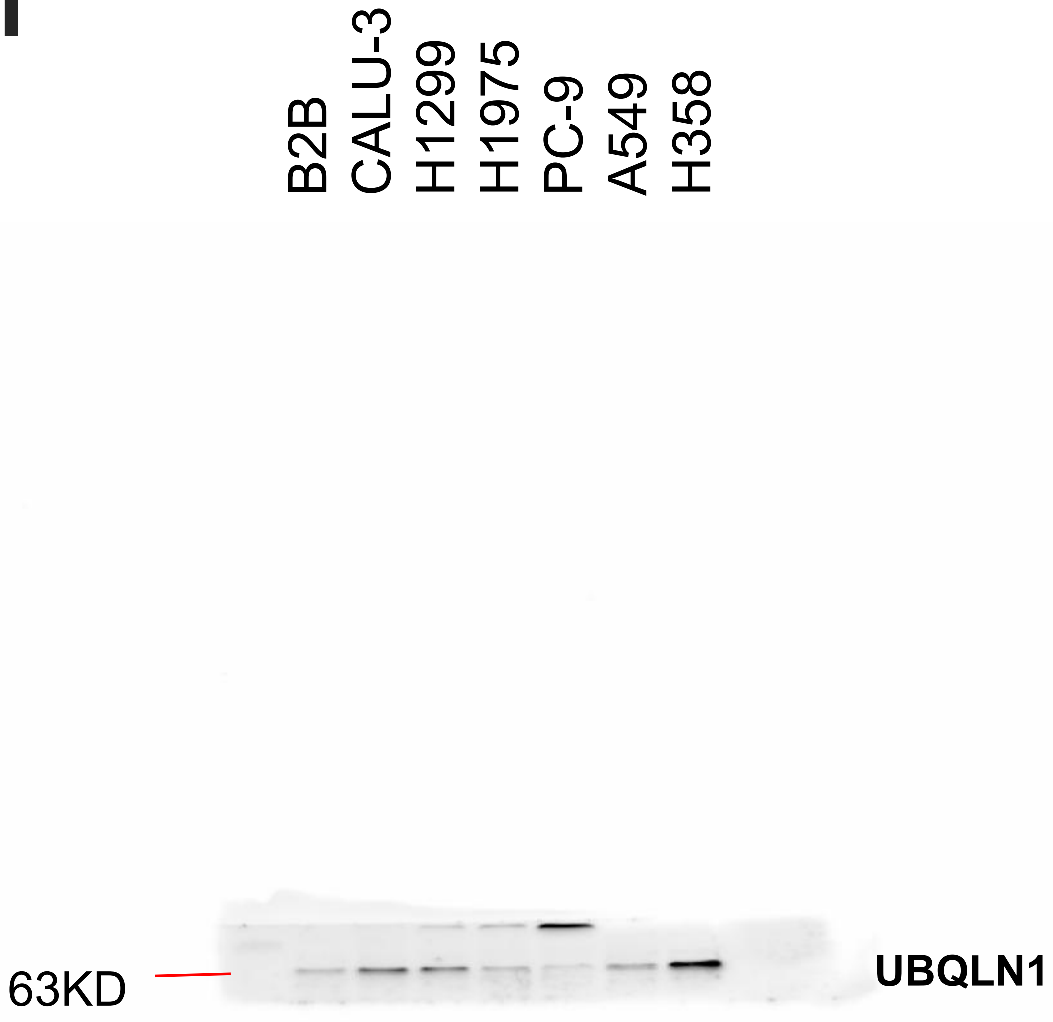

Fig7a-GAPDH

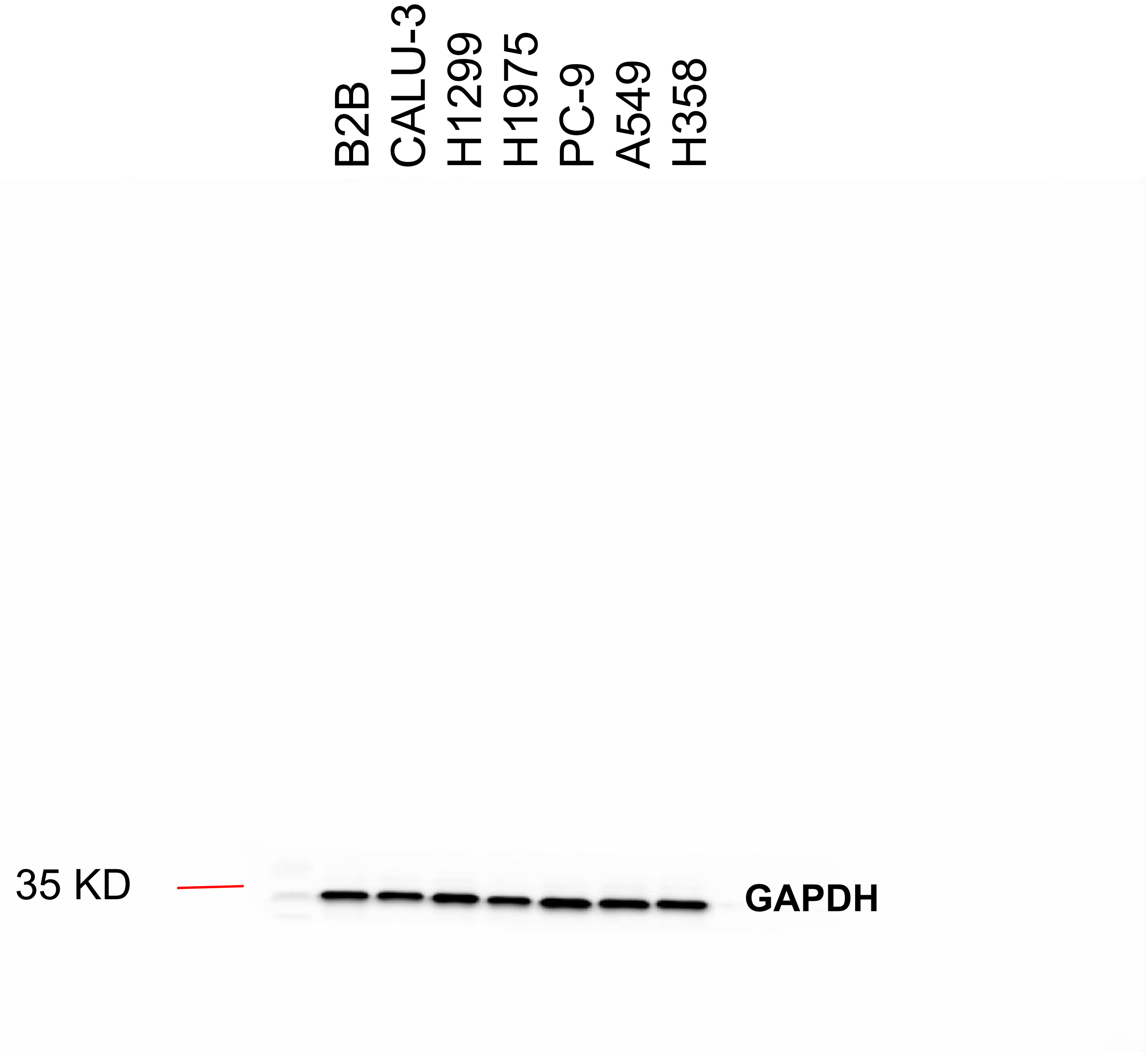

Fig7b-H358-UBQLN1

NC si-1 si-2 si-3

H358

63KD

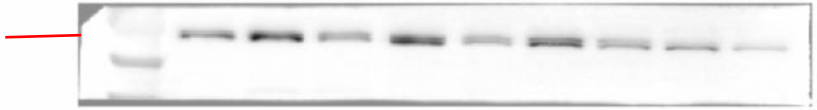

UBQLN1

Fig7b-H358-GAPDH

H358

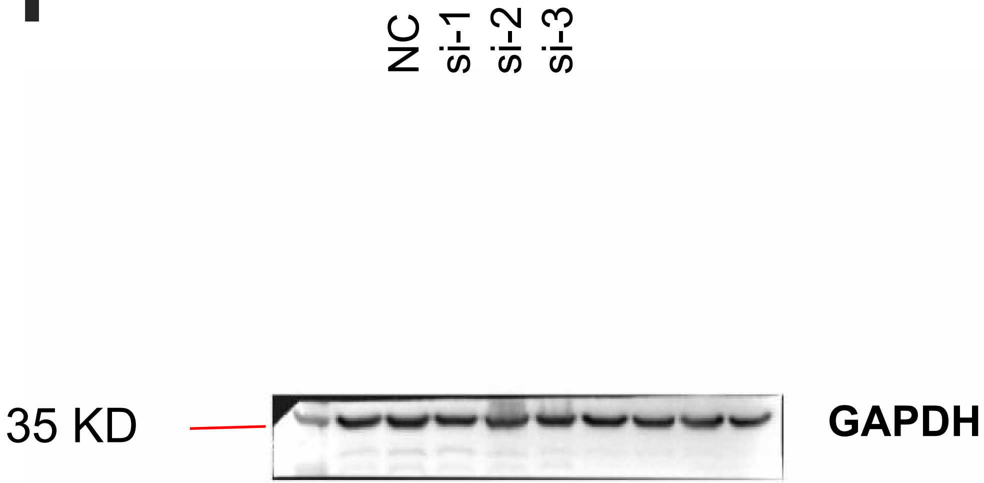

Fig7b-CALU-3-UBQLN1

CALU-3

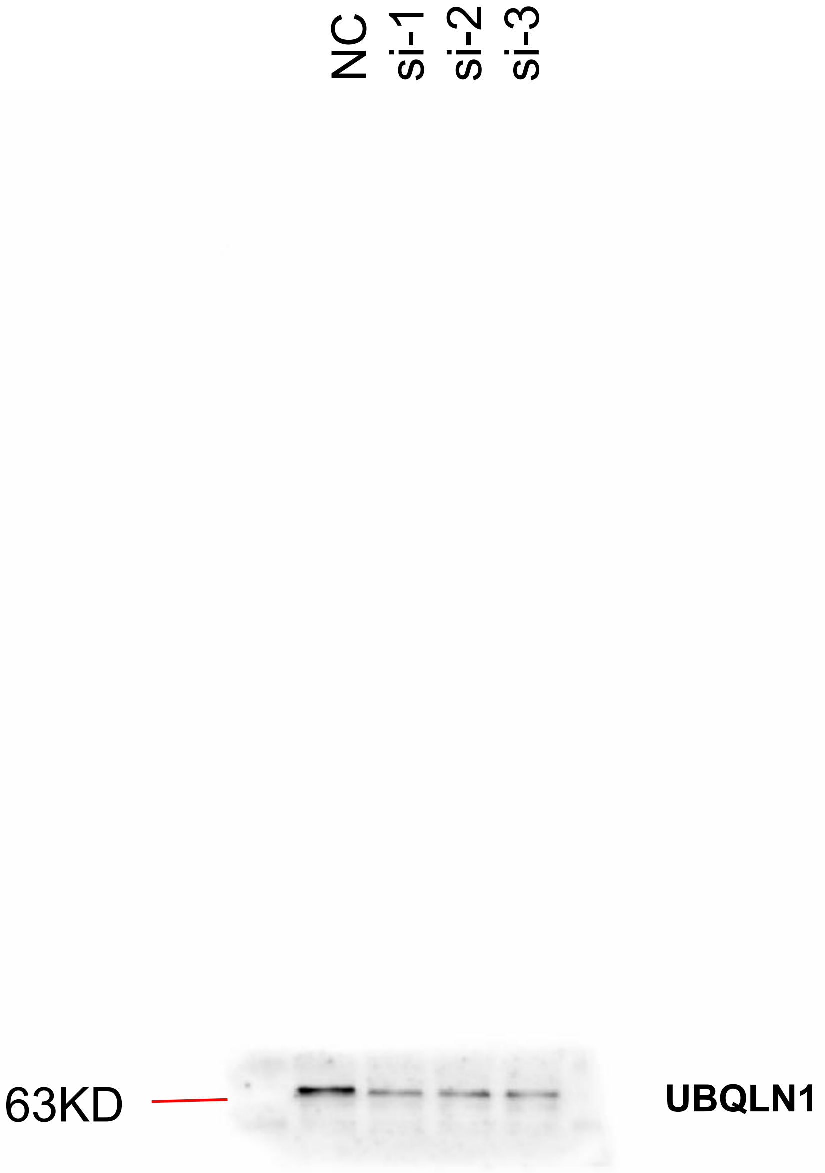

Fig7b-CALU-3-GAPDH

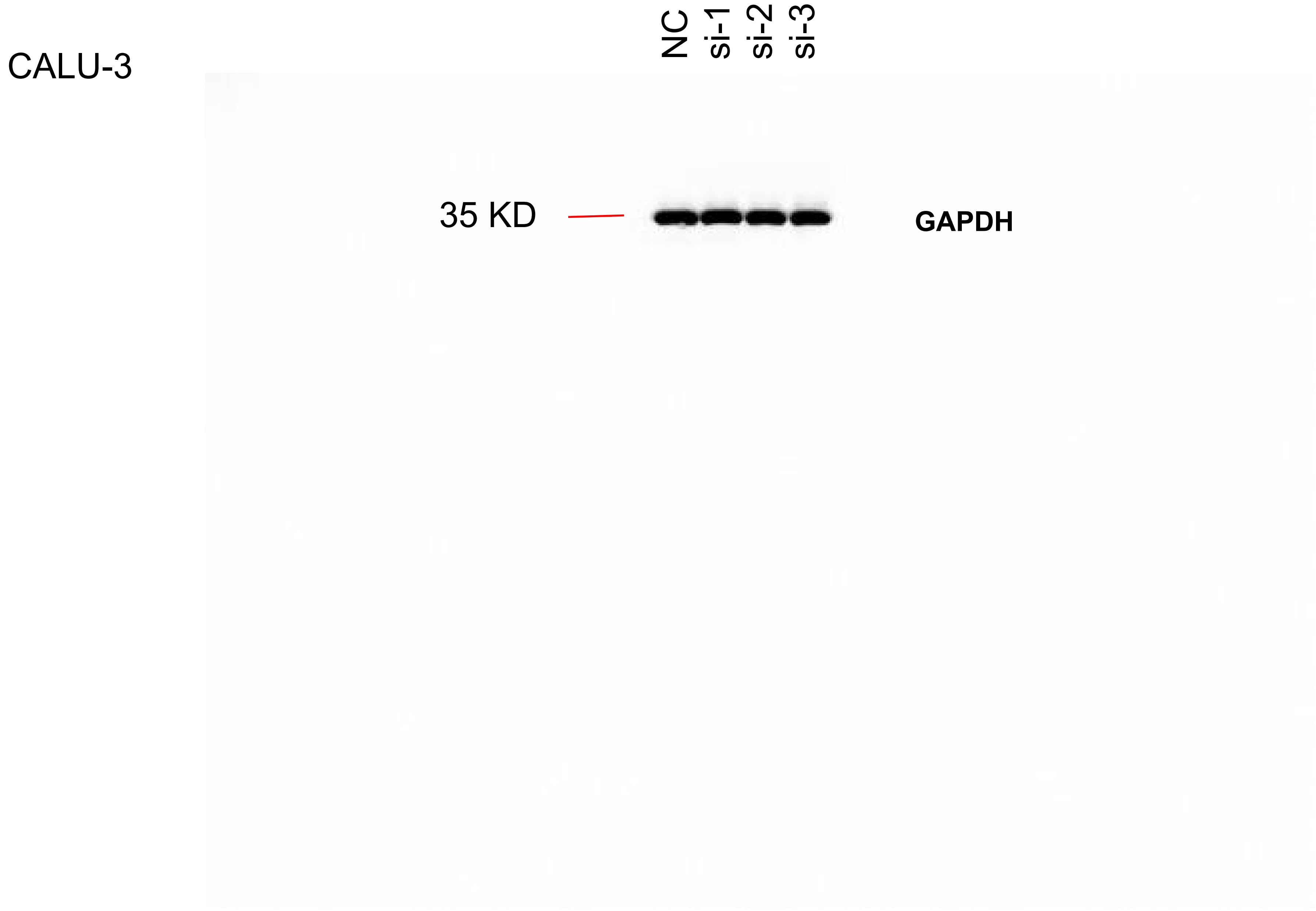

Figure S2-LC

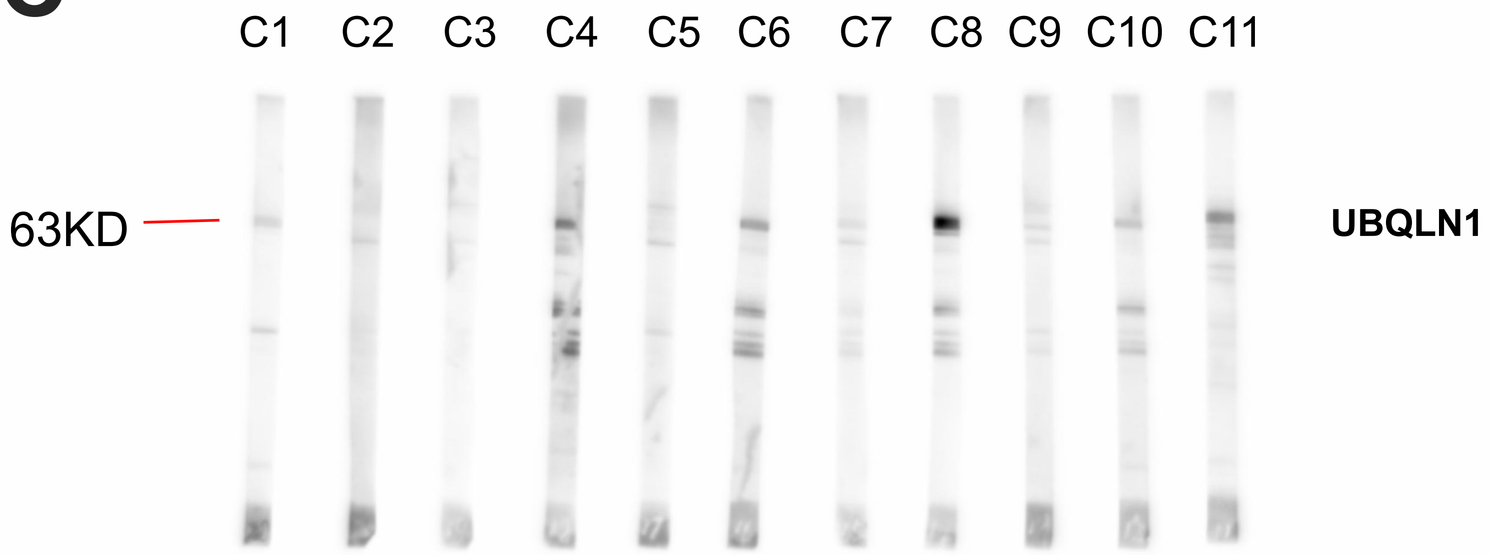

Figure S2-NC

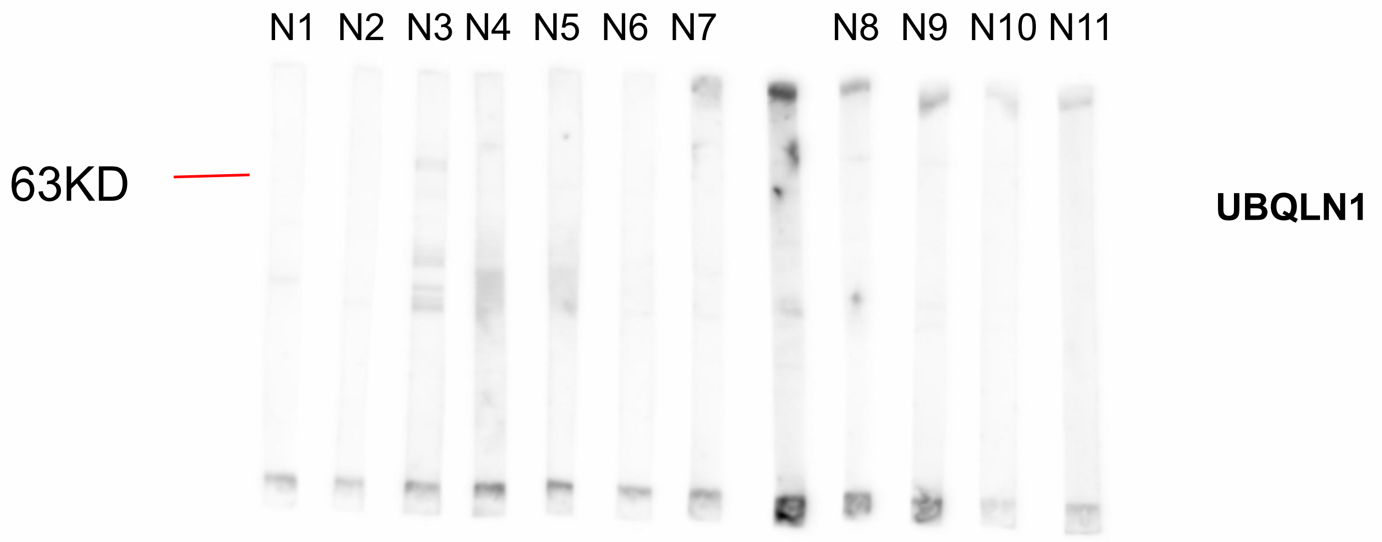

Supplement: Supplementary file 8 — Supplementary Material 8. [file 12885_2024_12019_MOESM8_ESM.pdf]
